# Ghrelin-induced neuronal NPY promotes brain metastasis in lung cancer patients with low BMI

**DOI:** 10.1038/s41467-025-60730-4

**Published:** 2025-07-01

**Authors:** Abhishek Tyagi, Shih-Ying Wu, Jee-Won Kim, Ravindra Pramod Deshpande, Kerui Wu, Eleanor C. Smith, Giuseppe L. Banna, Kounosuke Watabe

**Affiliations:** 1 Department of Cancer Biology, Wake Forest University School of Medicine, Winston-Salem, NC 27157 USA; 2 Nanoscience Department, Joint School of Nanoscience & Nanoengineering, Greensboro, NC 27401 USA; 3 Portsmouth Hospitals University NHS Trust, Faculty of Science and Health, School of Pharmacy and Biomedical Sciences, University of Portsmouth, Portsmouth, UK

**Keywords:** Non-small-cell lung cancer, Cancer metabolism, Metastasis, Tumour biomarkers

## Abstract

Obesity is a known risk factor for many cancers, yet recent studies reveal a paradoxical association between low body mass index (BMI) and increased brain metastasis in lung cancer—referred to as the “obesity paradox,” with unclear molecular mechanism(s). Here, we show a significantly higher incidence of brain metastasis in low-BMI lung cancer patients compared to those with high-BMI or other cancer brain metastasis in a pan-analysis of 7628 patients. Mechanistically, low BMI activates ghrelin-GHSR signaling, increasing neuronal neuropeptide Y (NPY) secretion, which promotes tumor metabolic reprogramming via NPY-Y5R, facilitating brain colonization. Elevated plasma ghrelin levels in cancer-free low-BMI subjects suggest its potential as a prognostic biomarker for predicting brain metastasis. Notably, targeting NPY-Y5R or reversing low BMI effectively suppresses brain metastasis, supporting its pro-metastatic role. These findings provide a strong rationale for developing targeted interventions to treat or prevent brain metastasis in lung cancer patients with low BMI.

## Abstract (web summary)

Low body mass index (BMI) is associated with increased brain metastasis risk in lung cancer patients. Here, the authors report that low-BMI increases neuronal secretion of neuropeptide Y (NPY), thereby activating the NPY receptor on lung tumor cells, leading to tumor metabolic reprogramming and brain metastasis.

## Introduction

Brain metastasis is the most common intracranial tumor, predominantly arising from lung (40–50%), breast (15–25%), and melanoma cancers (5–20%), with an annual incidence of 70,000 to 400,000 cases in the US^1,2^. Despite advancements in molecularly targeted therapies, immunotherapies, stereotactic radiosurgery, or traditional surgery^3^, the median survival ranges from 5 to 10 months^4^, underscoring the need to better understand this disease as a means to develop effective therapies. Importantly, lung cancer patients with brain metastasis have poor prognosis, high mortality rate, and frequent incidence of tumor recurrence due to associated clinical risk factors, including younger age, female gender, smoking, adenocarcinoma histology, karnofsky performance status (KPS), and the location and number of brain metastasis^5–8^. Notably, recent clinical epidemiological studies have revealed a positive association between low body mass index (BMI) and a two-fold increased risk of brain metastasis in lung cancer patients, even after adjusting for other predictive risk factors, leading to lower survival rates compared to patients with brain metastasis from other primary tumors^9–13^. How low-BMI contributes to an increased risk of lung cancer metastasis, preferentially in the brain compared to other organs, while simultaneously conferring a protective effect on other primary tumors, remains largely understudied. This paradoxical inverse relationship, referred to as the “obesity paradox,” necessitates further investigation to unravel the underlying mechanisms and nature of this phenomenon, particularly in the context of brain metastasis, which remains poorly understood. In this work, we address this question using a systematic approach, by analyzing the effects of low BMI-associated lung-to-brain metastasis. We show that low BMI induces neuronal NPY secretion and promotes brain metastasis in lung cancer by reprogramming cancer cell energy metabolism, thereby facilitating tumor progression.

## Results

### Low body mass index (BMI) promotes brain metastasis in lung cancer

**Fig. 1:**
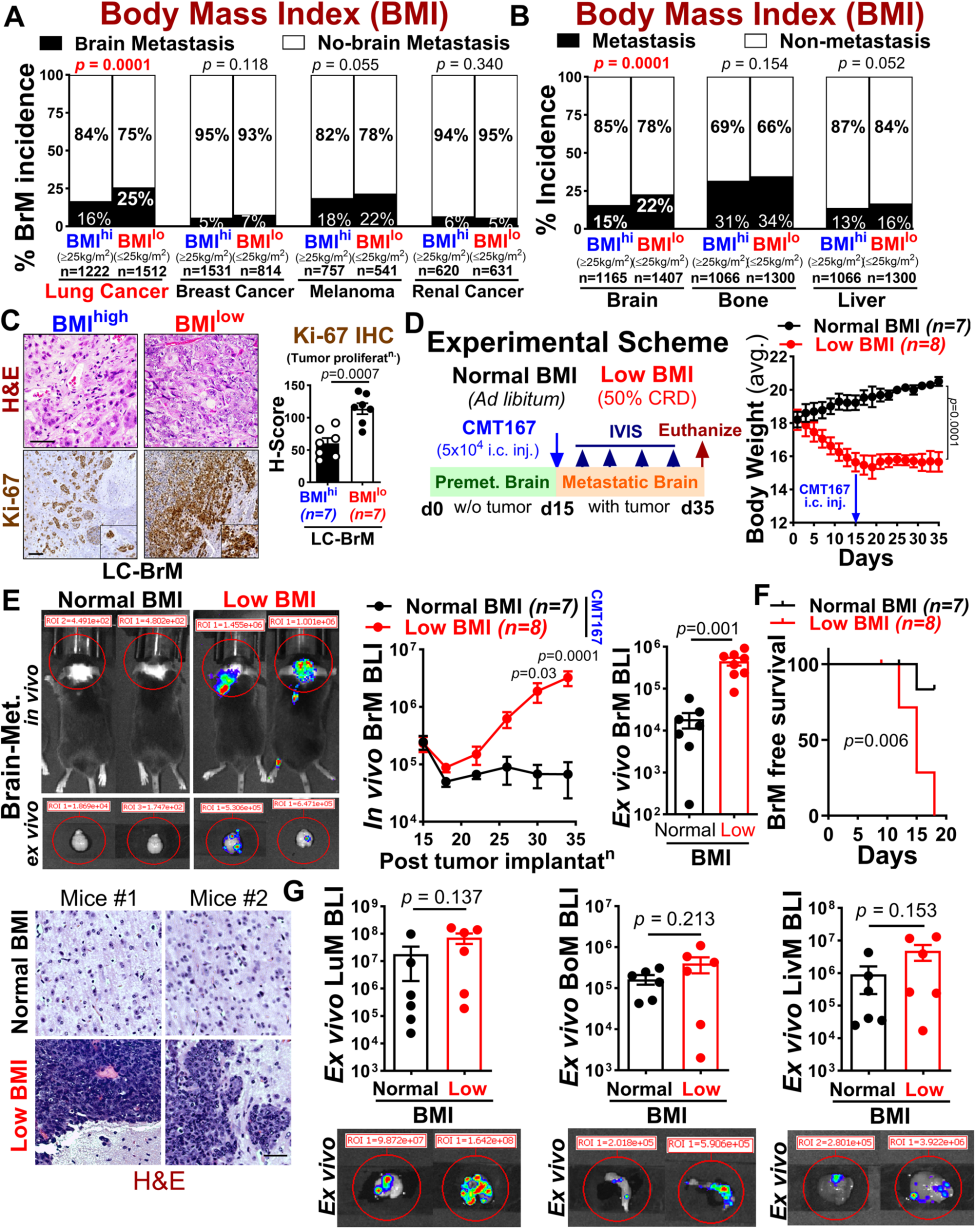
Low body mass index (BMI) is associated with increased brain metastasis in lung cancer. **A** Percentage of brain metastatic incidence across lung, breast, melanoma, and renal cancers (*n* = *7628*), stratified by BMI status. Patients with BMI < 25 were categorized as low BMI (BMI^low^) and those with BMI > 25 as high BMI (BMI^high^) (two-sided Fischer’s exact test). **B** Percentage of metastatic incidence in the brain, bone and liver within a lung cancer cohort (*n* = *2366*) comprising both low and high BMI patients (two-sided Fischer exact test). **C** Left: Representative hematoxylin and eosin (H&E) and Ki-67 immunohistochemistry images of brain metastatic tumor lesions from lung cancer patients, stratified by BMI [Scale bar: 100μm (H&E), 50μm (Ki-67); insert: 2 x Zoom]. Right: Dot plot showing quantification of Ki-67+ cells in brain metastatic tumor lesions from low and high BMI patients using H-score (*n* = *7* each, unpaired two-tailed t-test). **D** Left: Schematic of in vivo metastasis assay. Immune-competent C57BL/6 (female, 5–6 week) mice were fed with a standard or 50% calorie-restricted diet (CRD; 12-hr/dark phase) for 15 days to establish low BMI mice, followed by intracardiac injection (i.c.) of murine lung cancer cell line, CMT167 cells (5 × 10^4^). Right: Average body weight curve during the experiment (two-way ANOVA with Tukey’s test). **E** Top left: Representative in vivo (top) and ex vivo (bottom) BLI images of brain metastasis at endpoint. Middle/Right: In vivo and ex vivo BLI quantification of brain metastasis in normal (*n* = *7*) and low BMI (*n* = *8*) mice at endpoint (two-way ANOVA with Tukey’s test (in vivo); unpaired two-tailed t-test (ex vivo). Bottom: Representative H&E images of brain metastatic tumor lesions from normal and low BMI mice (Scale bar: 50 μm). **F** Kaplan–Meier analysis of brain metastasis-free survival in normal (*n* = *7*) and low BMI (*n* = *8*) mice [log-rank (Mantel–Cox test)]. **G** Representative ex vivo BLI images and quantification of lung, liver and bone metastasis in normal and low BMI mice (*n* = *6/*group) at endpoint (unpaired two-tailed t-test). All data are mean ± S.E.M. Source data are provided as a Source Data file.

To understand the mechanism by which low BMI promotes brain metastasis preferentially in lung cancer compared to other cancer types, we performed a retrospective pan-analysis of multiple patient cohorts with brain metastasis from lung, breast, melanoma and renal cancers with their BMI status, comprising a total of 7628 cancer patients^14–24^. We found a significant association between low BMI and a higher incidence of brain metastasis in lung cancer, in contrast to breast, melanoma and renal cancer (Fig. 1A). Notably, within a clinical cohort of metastatic lung cancer patients (*n* = 2366)^14–16,24^, low BMI exhibited a significant association with brain metastasis compared to extracranial metastatic sites including bone and liver (Fig. 1B), and is also significantly associated with poor brain-metastasis free survival in a clinical cohort of metastatic lung cancer patients (*n* = 513)^25^ (Supplementary Fig. 1A). Importantly, patients with lung cancer brain metastasis showed no significant difference with other clinical factors, including gender, race, smoking, *EGFR* mutation except for age (Supplementary Fig. 1B)^26–29^. To further gain insight into the underlying mechanism of low BMI-induced brain metastasis, we utilized clinical samples of brain metastatic lung cancer patients with BMI status and assessed Ki-67 immunoreactivity by immunohistochemistry. We found that brain metastatic patients with low BMI exhibited a higher number of Ki-67 positive tumor cells compared to those with high BMI, indicating an increase in tumor growth in brain metastatic lung cancer patients with low BMI (Fig. 1C). This contrasts with previous studies linking high BMI (obesity) with more aggressive tumor growth and increased cancer mortality in other cancer types^11,12,30,31^. To investigate how BMI impacts brain metastasis in vivo, we established a low-BMI mouse model through a regimen of 50% calorie restriction combined with time-restricted feeding to mimic human low BMI conditions (Supplementary Fig. 1C–E, and Supplementary Table 1) and examined its role in experimental metastasis by implanting CMT167 and LL2 mouse lung cancer cells in syngeneic C57BL/6 mice via intracardiac injection (i.c.) (Fig. 1D) followed by monitoring the metastatic burden by bioluminescence imaging (BLI) (Fig. 1E). We found that low BMI significantly increased the brain metastatic burden by more than 10-fold and exhibited significantly reduced brain-metastasis free survival compared to normal BMI (Fig. 1E–F, and Supplementary Fig. 1F–H). Importantly, low BMI has no significant impact on extracranial metastasis in comparison to normal BMI (Fig. 1G), consistent with our clinical observation. Taken together, these results strongly imply that low BMI plays a critical role in creating a favorable brain metastatic niche in lung cancer metastasis.

### Low BMI-mediated ghrelin activates neurons that promote tumor growth via NPY

**Fig. 2:**
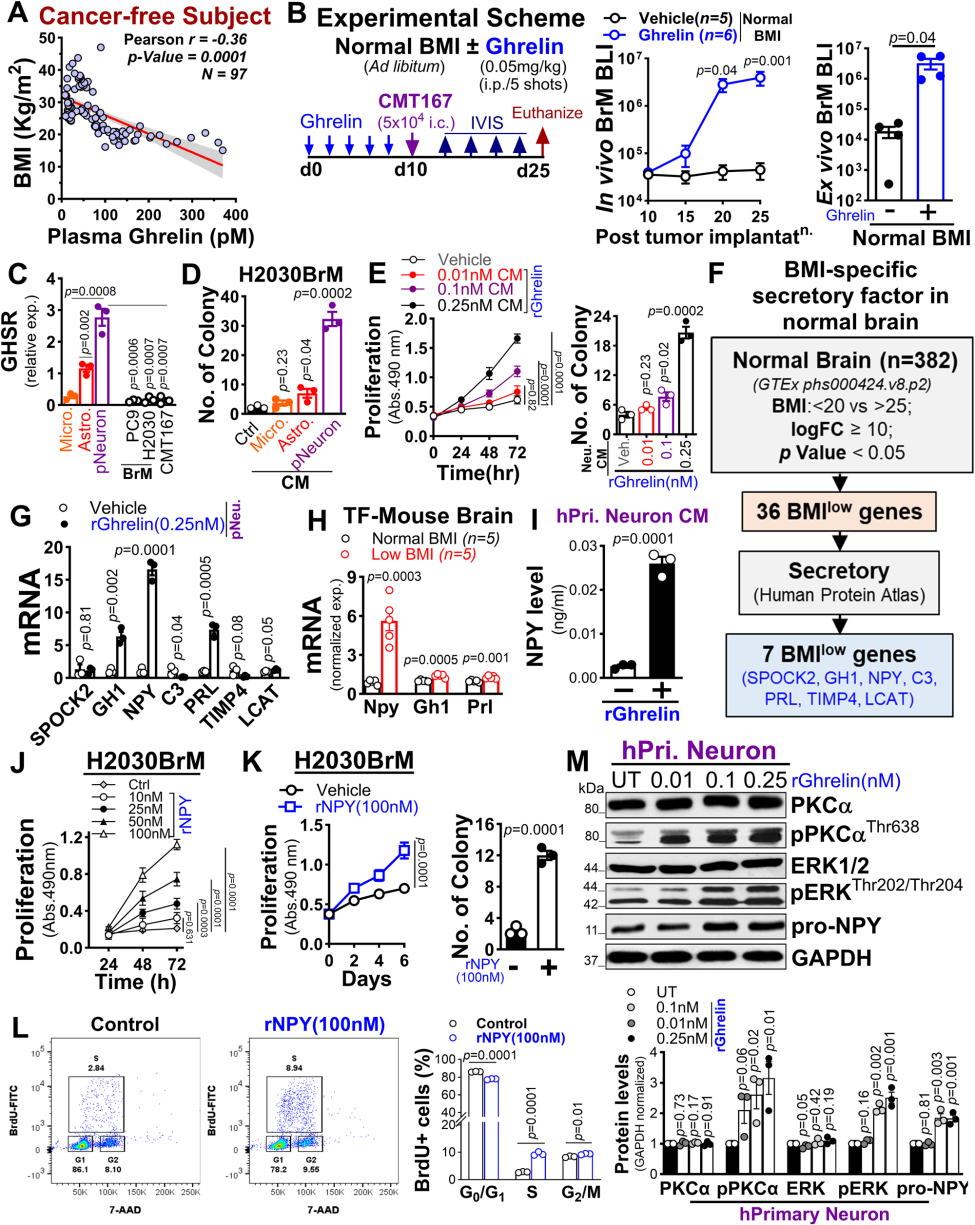
Low BMI promote tumor growth via ghrelin activated neuronal NPY. **A** Pearson correlation between BMI and plasma ghrelin levels in cancer-free subjects (*n* = *97*) using a publicly available dataset (two-tailed; 95% CI shaded). **B** Left: Schematic of in vivo metastasis assay. Female C57BL/6 mice (5-6 weeks) were fed with a standard diet and administered ghrelin (0.05 mg/kg; i.p.; 5 shots) for 10 days before CMT167 cells injection (5 × 10^4^; i.c.). In vivo (middle) and ex vivo (right) BLI quantification of brain metastasis in control (*n* = *5*) and ghrelin-treated normal BMI mice (*n* = *6*) [two-way ANOVA with Tukey’s test (in vivo); unpaired two-tailed t-test (ex vivo)]. **C** Relative *GHSR* expression in human brain cells (HMC3, UC1, primary neurons) and lung cancer cell lines (PC9BrM, H2030BrM, CMT167) by qRT-PCR, normalized to *β-Actin* (*n* = *3* independent experiments, unpaired two-tailed t-test). **D** Colony formation of H2030BrM cells treated with or without CM from indicated brain cells (*n* = *3* independent experiments, unpaired two-tailed t-test). **E** MTS (left) and colony (right) assays of H2030BrM cells treated with or without CM from ghrelin-treated primary neurons (0.01 nM-0.25 nM; *n* = *3* independent experiments, two-way ANOVA with Tukey’s test). **F** Strategy to identify low BMI-specific brain secretory factors using GTEx and HPA database (*n* = *382*). **G** qRT-PCR of indicated genes in primary neurons treated with or without rGhrelin (0.25 nM, 12 h, normalized to *β-Actin*; *n* = *3* independent experiments, unpaired two-tailed t-test). **H** Expression of *Npy*, *Gh1* and *Prl* in brains of normal and low-BMI mice (Fig. 1D) by qRT-PCR (normalized to *β-Actin*; *n* = *3* independent experiments, unpaired two-tailed t-test). **I** NPY protein levels in CM from untreated and ghrelin-treated (0.25 nM) neurons by ELISA (*n* = *3* independent experiments, unpaired two-tailed t-test). **J**,** K** MTS and colony assays of H2030BrM cells treated with rNPY [10–100 nM, short-term (J); 100 nM, long-term (K)]; (J: *n* = *3* independent experiments, two-way ANOVA with Tukey’s test; K: *n* = *3* independent experiments, unpaired two-tailed t-test). **L** Dot plot (left) and quantification (right) of BrdU-based cell cycle analysis of H2030BrM cells treated with or without rNPY (100 nM; *n* = *3* independent experiments, unpaired two-tailed t-test). **M** Western blot (upper) and ImageJ quantification (lower) of GHSR signaling proteins in neurons treated with or without rGhrelin (0.01-0.25 nM, 24 h; normalized to GAPDH; *n* = *3* independent experiments, unpaired two-tailed t-test). All data are mean ± S.E.M. Source data are provided as a Source Data file.

What factors drive increased brain metastasis in patients with low BMI is an intriguing question. Prior studies have shown that plasma ghrelin, an orexigenic peptide regulating growth hormone secretion, food intake, and body weight through the growth hormone secretagogue receptor (*GHSR*)^32–39^, is elevated in both lean and healthy individuals undergoing fasting or calorie restriction but decreased in obese individuals, suggesting a compensatory role in response to low BMI^40–44^. We also analyzed cancer-free subjects with known BMI data^43,45,46^ and found a significant inverse correlation between high plasma ghrelin levels and low BMI compared to those with high BMI (Fig. 2A). Therefore, to understand the functional relationship between ghrelin and its potential impact on low BMI-induced brain metastasis, we treated normal BMI mice with ghrelin or administered a pharmacological GHSR inhibitor to low BMI mice. Experimental metastasis was assessed by intracardiac injection (i.c.) of CMT167 or LL2 mouse lung cancer cells into syngeneic C57BL/6 mice (Fig. 2B, and Supplementary Fig. 2A–B). We found that ghrelin treatment significantly increased brain metastatic burden by over 10-fold, whereas GHSR inhibition reduced brain metastasis by more than 20-fold. These treatments also significantly affected survival, with ghrelin decreasing and GHSR inhibition increasing brain metastasis-free survival compared to the controls (Supplementary Fig. 2A–C). Notably, neither treatment impacted extracranial metastasis compared to controls (Supplementary Fig. 2A–D). Next, we examined the expression of the ghrelin receptor in various brain cell types as well as in parental and brain-tropic lung cancer cells. An enriched overexpression of *GHSR* receptor was found in neurons, consistent with a previous study^34^, in contrast to other brain cell types or lung cancer cells, which exhibited low to negligible levels of *GHSR* expression (Fig. 2C, and Supplementary Fig. 3A). Furthermore, treating lung cancer cells with a physiological concentration of recombinant ghrelin (rGhrelin; 0.1nM-0.25 nM) showed no discernible effect on cancer cell proliferation and growth in a dose-dependent manner (Supplementary Fig. 3B). Conversely, conditioned media (CM) derived from primary neurons significantly increased cancer cell growth compared to CM from other brain cell types, such as astrocytes and microglia (Fig. 2D). To understand how neurons impact tumor cell proliferation and growth under low BMI conditions, we examined the effect of CM from primary neurons pre-exposed to physiological concentration of recombinant ghrelin (0.1nM-0.25 nM) on tumor cells. We found that CM from primary neurons pretreated with increasing doses of recombinant ghrelin protein significantly enhanced tumor cell proliferation and growth (Fig. 2E, and Supplementary Fig. 3C). To gain deeper insights into how low-BMI influences the brain secretome in response to elevated plasma ghrelin, we analyzed publicly accessible RNA-seq data from the normal human brain with BMI status (*n* = *382*) from the GTEx (Genotype-Tissue Expression; dbGaP accession number phs000424.v8), in conjunction with the Human Protein Atlas (HPA) database^47^, and found 7 low BMI-specific secretory genes in the brains of cancer-free subjects with low BMI (Fig. 2F, and Supplementary Table 2**;** unpaired two-tailed t-test). Further validation in human primary neurons pretreated with recombinant ghrelin showed a significantly enriched expression of neuropeptide Y (*NPY*) compared to other genes (Fig. 2G), and the expression was further increased with escalating doses of recombinant ghrelin (0.1nM-0.25 nM), primarily within neurons as opposed to other brain cell types (Supplementary Fig. 3D–E). Importantly, similar results were observed in tumor-free low BMI mice (from Fig. 1; panel D) as well as in normal brain tissue and brain metastatic tissue from H2030BrM xenografts, in contrast to normal BMI mice, non-brain tissue or primary lung cancer (Fig. 2H, Supplementary Fig. 3F–G). Additionally, conditioned media (CM) from ghrelin-treated primary neurons showed significantly higher NPY levels compared to controls or cancer cells (Fig. 2I, and Supplementary Fig. 3H). Notably, when lung cancer cells were treated with recombinant NPY (rNPY), they showed a dose-dependent increase in cell proliferation, growth kinetics and cell cycle progression. These results suggest that the tumor-promoting effect of low BMI is likely mediated by ghrelin-activated neurons, which secrete NPY with a potential growth-promoting role in tumor cells (Fig. 2J–L, and Supplementary Fig. 3I). Next, to elucidate the molecular pathway responsible for ghrelin-induced NPY secretion, we focused on the PKCα/ERK pathway, previously implicated in neuronal NPY regulation in a ghrelin-dependent manner^48,49^. We observed a ghrelin-specific, dose-dependent increase in NPY protein levels, accompanied by an elevated expression of its upstream effector protein (PKCα/pERK) in primary neurons (Fig. 2M). Notably, this expression was abrogated in primary neurons by a lower dose of a pharmacological ERK1/2 inhibitor (SCH772984), in contrast to cancer cells, which exhibited cell toxicity and growth suppression at higher doses (Supplementary Fig. 3J–L), providing further confirmation of the PKCα/ERK axis as the key signaling pathway responsible for ghrelin-mediated neuronal NPY secretion via *GHSR*. Collectively, these results indicate the pro-tumorigenic role of neuronal NPY in promoting brain metastasis under low-BMI conditions, distinct from its physiological role in regulating feeding behavior and maintaining energy homeostasis.

### Neuronal NPY promotes brain metastasis via NPY5R (Y5R) signaling

**Fig. 3:**
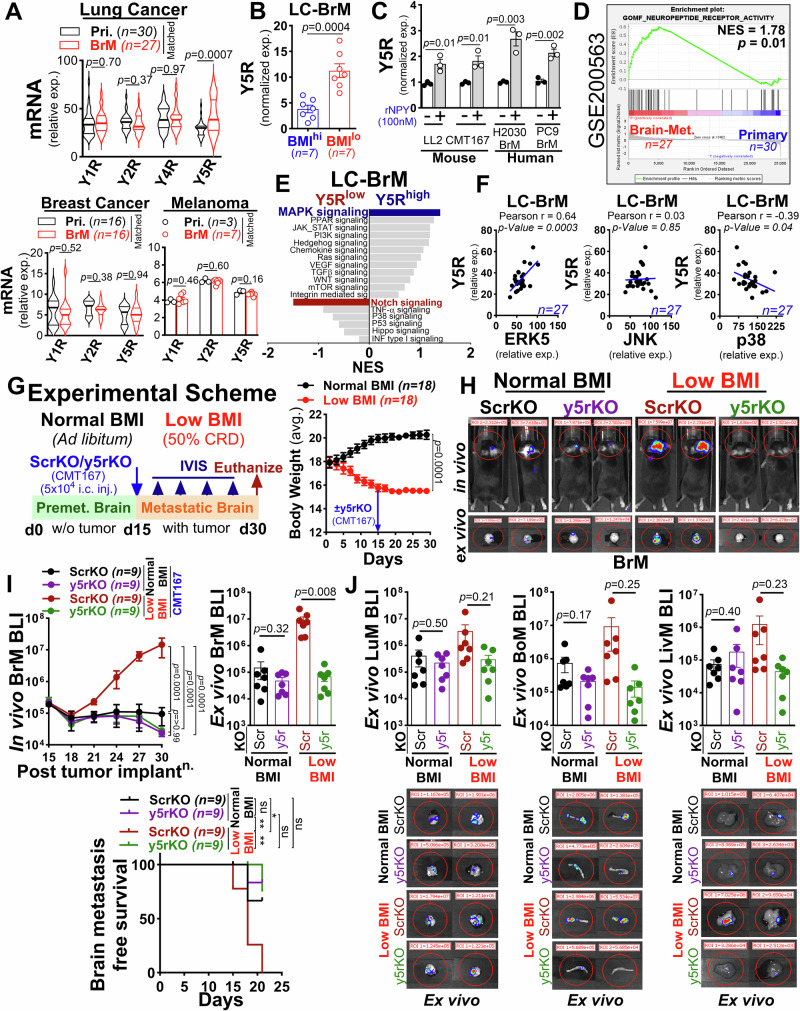
Low BMI-mediated neuronal NPY promotes brain metastasis via Y5R signaling. **A** Violin and dot plots analysis showing NPY receptor expression in matched brain metastatic samples from lung, breast, and melanoma cancer patients using GEO datasets (GSE200563 - upper; GSE125989 and GSE44660 -lower panels; unpaired two-tailed t-test). **B** qRT-PCR analysis of *Y5R* expression in brain metastatic tissue from lung cancer patients with low and high BMI (*n* = *7/*group), normalized to *β-Actin* (unpaired two-tailed t-test). **C** qRT-PCR analysis of *Y5R* expression in human and mouse lung cancer cell lines treated with 100 nM rNPY, normalized to *β-Actin* (*n* = *3* independent experiments, unpaired two-tailed t-test). **D** Gene Set Enrichment Analysis (GSEA) of neuropeptide receptor activity in matched lung (*n* = *30*) and brain metastatic tumors (*n* = *2*7) from GSE200563, using pre-ranked MSigDB v5.1gene sets. Enrichment scores generated by random forest-based ranking; *p*-values indicate statistical significance. **E** Bar plot showing enriched signaling pathways in Y5R^high^ and Y5R^low^ lung cancer brain metastasis (GSE200563). **F** Pearson correlation between *Y5R* and *MAPK* family members in brain metastatic patients (*n* = *27*, GSE200563; two-tailed); *Y5R-ERK5* (*p* = 0.0003), *Y5R-JNK* (*p* = 0.04) and *Y5R-p38* (*p* = 0.853). **G** Left: Schematic of in vivo metastasis assay. Female C57BL/6 mice (5–6 weeks) were fed with a standard or 50% CRD (12-hr/dark phase) for 15 days before ScrKO and y5rKO CMT167 cells injection (5 × 10^4^; i.c.). Right: Average body weight curve (two-way ANOVA with Tukey’s test). **H** Representative in vivo (top) and ex vivo (bottom) BLI images of brain metastasis at endpoint. **I** Top: In vivo (*n* = *9/*group; left) and ex vivo *(n* = *7/*group; right) BLI quantification of brain metastasis in ScrKO and y5rKO implanted mice [two-way ANOVA with Tukey’s test *(*in vivo); unpaired two-tailed t-test (ex vivo)]. Bottom: Kaplan–Meier analysis of brain metastasis-free survival in ScrKO and y5rKO implanted mice [*n* = *9/*group; log-rank (Mantel–Cox test)]. **J** Representative ex vivo BLI images and quantification of extracranial metastasis in ScrKO and y5rKO implanted mice (*n* = *7/*group) at endpoint (unpaired two-tailed t-test). All data are mean ± S.E.M. Source data are provided as a Source Data file.

To gain further insight into the functional role of low BMI-induced NPY in brain metastasis, we queried clinical GEO cohort data of lung^50^, breast^51^, and melanoma^52^ cancers, comprising matched primary and brain metastasis samples to assess NPY receptor expression patterns. We found that brain metastatic lesions of lung cancer exhibited significantly higher expression of the *NPY5R* (*Y5R*) compared to other NPY receptors, and the expression of *Y5R* was selectively enriched in metastatic lung cancer, in contrast to metastatic breast and melanoma cancer (Fig. 3A). Additionally, increased expression of Y5R is associated with poor overall survival in lung cancer patients, attributed to its elevated levels in metastatic lung tumors compared to normal and primary lung tumors. In contrast, in breast cancer, *Y5R* expression is correlated with better overall survival, with higher expression observed in normal and primary tumors compared to metastatic breast tumors (Supplementary Fig. 4A). To further validate this observation, we analyzed multiple GEO cohort datasets, including clinical and preclinical samples of lung, breast, and melanoma metastatic tumors, as well as primary tumor xenografts (GSE123904 /GSE48433 /GSE14020/GSE50493)^53–56^. We found that *Y5R* was selectively expressed in metastatic tumors, primarily in the brain, in lung cancer, in contrast to metastatic tumors from breast and melanoma cancers (Supplementary Fig. 4B). As the Y5R has recently been reported to play a critical role in tumor progression^57,58^, we next examined its expression in brain cells and lung cancer cells. We found that cancer cells exhibited a higher level of *Y5R* expression compared to brain cells, except neurons that showed basal expression as reported previously^59,60^ (Supplementary Fig. 4C). We then examined clinical brain metastatic samples from lung cancer patients with BMI history. We observed significantly higher *Y5R* expression in lung cancer brain metastatic patients with low BMI compared to those with high BMI (Fig. 3B). Similar results were obtained in murine and human lung cancer cell lines, both at transcript and protein levels, and its expression was significantly upregulated in the cancer cells in the presence of recombinant NPY (Fig. 3C, and Supplementary Fig. 4D). Additionally, we analyzed RNA-seq data and performed Gene Set Enrichment Analysis (GSEA)^61^ to evaluate neuropeptide receptor activity in matched primary and brain metastatic tissues obtained from a clinical study of lung cancer patients^50^. We found significantly elevated neuropeptide receptor signaling in brain metastatic tumors compared to primary tumors (Fig. 3D), suggesting that neuropeptide receptor activity may play a crucial role in governing tumor cell aggressiveness and metastatic progression. NPY peptides and their receptors are known to influence cancer progression through distinct signaling pathways^62^, but the underlying molecular mechanisms remain elusive. Therefore, we analyzed existing RNA-sequencing data of brain metastatic tumors from a lung cancer clinical cohort^50^ and performed GSEA for oncogenic pathways by stratifying brain metastatic patients based on their *NPY* receptor expression. We found that brain metastatic patients with a high *NPY* receptor level (*Y5R*^*high*^) showed activated MAPK signaling signatures, while patients with a low NPY receptor level (*Y5R*^*low*^) displayed signatures associated with the Notch pathway (Fig. 3E). This indicates potential divergent roles of Y5R signaling in brain metastasis. In support of this finding, we noted a positive correlation between *Y5R* and *ERK5* expressions compared to *p38* and *JNK*, members of the MAPK signaling pathway in brain metastatic patient cohort (Fig. 3F). To further characterize the role of the Y5R in promoting low BMI-induced neuronal NPY-mediated tumor growth, we utilized CRISPR/Cas9 technology to knockout *Y5r* in a murine CMT167 lung cancer model. Two independent clones with a stable *Y5r* knockout (*Y5r*KO) were established, as described in the “Methods” section. The knockout of *Y5r* did not affect cancer cell growth upon recombinant NPY treatment, as compared to scrambled knockout (ScrKO) control cells (Supplementary Fig. 4E–G). We then performed an in vivo experimental metastasis by intracardiac injection of luciferase-expressing ScrKO or Y5rKO cancer cells into syngeneic C57BL/6 mice with normal or low BMI conditions. We found that loss of the Y5r significantly decreased the brain metastatic tumor growth and resulted in a significant increase in brain-metastasis free survival in low BMI mice compared to the control mice implanted with scrambled knockout cancer cells (Fig. 3G–I). The *Y5r* knockout did not show a significant impact on extracranial metastasis under low BMI conditions as compared to the control mice with normal BMI (Fig. 3J). These results are consistent with our clinical observations, highlighting the significant impact of low BMI on brain metastasis. Overall, these results suggest that enhanced neuronal activity under low BMI conditions promotes cancer cells proliferation and growth through the NPY/Y5R signaling axis in the brain during the early stages of metastatic colonization.

### Neuronal NPY promotes metabolic reprogramming in tumor cells by upregulating lipogenesis via Y5R/ERK5/SREBP2/FASN pathway

**Fig. 4:**
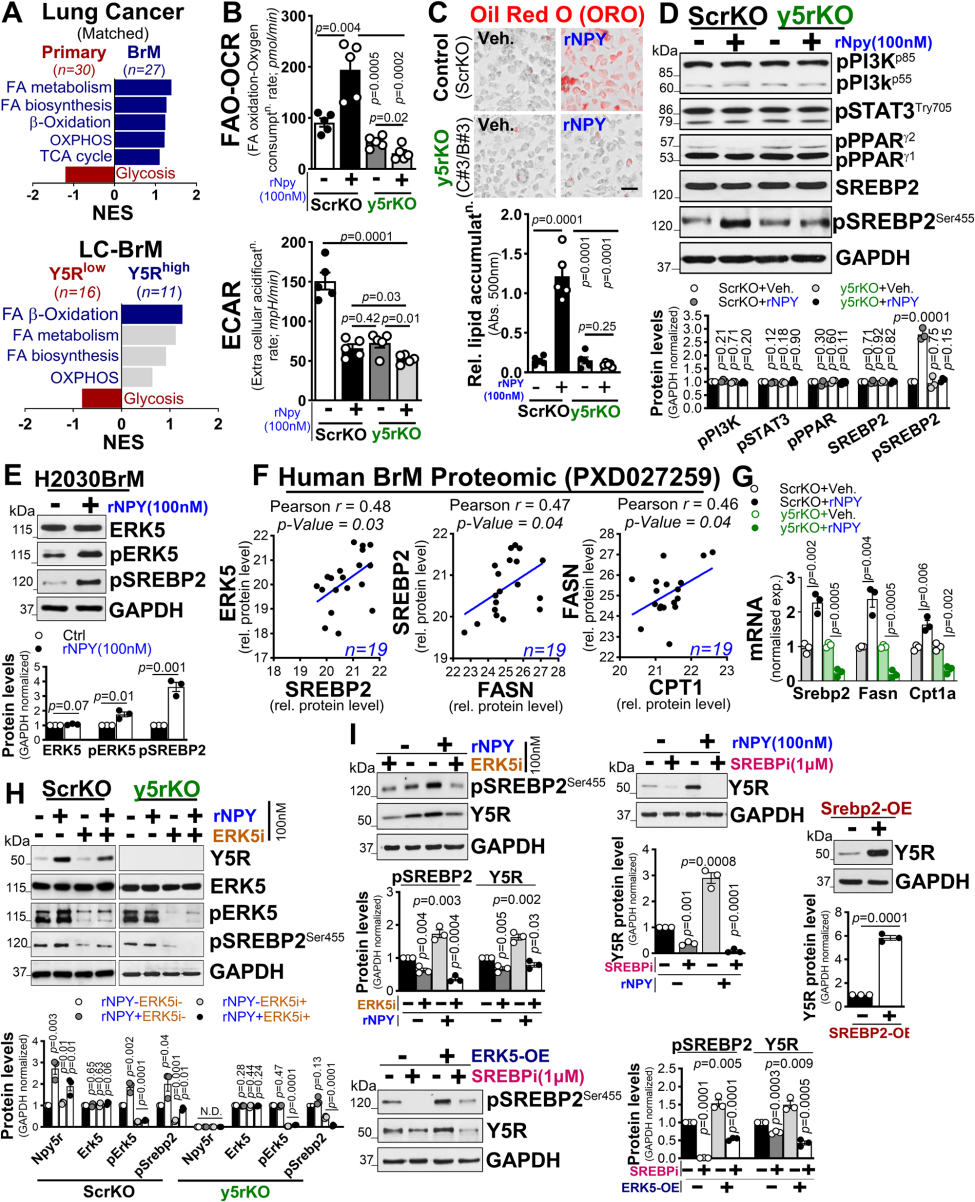
Low BMI-induced neuronal NPY upregulates lipogenesis via the Y5R/SREBP2/FASN signaling axis to fuel tumor cell growth. **A** Metabolic GSEA was performed in matched primary lung (*n* = *30*) and brain metastatic (*n* = *27*) tumors (top) and between Y5R^high^ and Y5R^low^ brain metastatic lesions (bottom, GSE200563). Gene sets were derived from Gene Ontology (GO:0006635; GO:0006099; GO:0061621; GO:0046949, GO:0006119, GO:0006631)^153^. **B** FAO-driven oxygen consumption (OCR) and glycolytic rate (ECAR) were measured in ScrKO and y5rKO CMT167 cells pretreated with or without rNPY (100 nM) (*n* = *5* independent experiments, unpaired two-tailed t-test). **C** Representative images (top) and quantification (bottom) of Oil Red O staining showing lipid accumulation in ScrKO and y5rKO lung cancer cells treated with or without rNPY (100 nM, 12 h; Scale bar: 50 μm; *n* = *5* independent experiments; unpaired two-tailed t-test). **D** Top: Western blot of SREBP2 and Y5R^high^-enriched key oncogenic pathways in ScrKO and y5rKO CMT167 cells treated with or without rNPY (100 nM, normalized to GAPDH; *n* = *3* independent experiments, unpaired two-tailed t-test). Bottom: Band intensity quantification by ImageJ (unpaired two-tailed t-test). **E** Top: H2030BrM cancer cells treated with or without rNPY (100 nM) and immunoblotted for activated ERK5 and SREBP2, normalized to GAPDH (*n* = *3* independent experiments, unpaired two-tailed t-test). Lower: Band intensity quantification by ImageJ (unpaired two-tailed t-test). **F** Pearson correlation between ERK5-SREBP2, *p* = 0.036; SREBP2-FASN, *p* = 0.041 and FASN-CPT1, *p* = 0.047 in lung cancer brain metastatic patients using a proteomic dataset (PXD027259; *n* = *19;* two-tailed)^67^. **G** Expression of *Srebp2*, *Fasn* and *Cpt1a* in ScrKO and y5rKO CMT167 cells treated with or without rNPY (100 nM), by qRT-PCR, normalized to *β-Actin* n (*n* = *3* independent experiments, unpaired two-tailed t-test). **H** Top: Cells from panel G were further immunoblotted for Y5R, pERK5 and SREBP2 in the presence or absence of rNPY (100 nM) or ERK5 inhibitor (100 nM each), GAPDH normalized (*n* = *3* independent experiments, unpaired two-tailed t-test). Bottom: Band intensity quantification by ImageJ (unpaired two-tailed t-test). **I** Top: LL2 cells were transfected with or without ERK5/SREBP2 expression plasmids or treated with or without SREBP (Fatostatin HBr) or ERK5 inhibitor (ERK5-IN-1), in presence or absence of rNPY (100 nM) were immunoblotted for activated SREBP2 and Y5R, GAPDH normalized (*n* = *3* independent experiments, unpaired two-tailed t-test). Bottom: Band intensity quantification by ImageJ (unpaired two-tailed t-test). All data are mean ± S.E.M. Source data are provided as a Source Data file.

Emerging studies on neuropeptide receptor signaling have highlighted its physiological role in the central regulation of metabolic homeostasis^63^. In addition, recent evidence also indicates that differences in nutrient availability in brain tissue relative to other tissue can necessitate metabolic adaptation or competition by cancer cells for growth in the brain^64,65^. However, the intricate mechanisms underlying activated Y5R influence on tumor cell metabolism remain elusive. To address this question, we queried existing RNA-seq. data of matched primary and brain metastatic tissues from a clinical cohort of lung cancer^50^ and evaluated major metabolic pathways by GSEA. We found that fatty acid metabolism was highly enriched in brain metastatic tumors, while glycolytic pathways were activated in primary tumors (Fig. 4A). Similar results were obtained in another clinical cohort of patients with lung cancer brain metastasis (Supplementary Fig. 5A). Interestingly, brain metastatic patients with a high NPY receptor level (Y5R^high^) showed activated fatty acid β-oxidation signatures, whereas patients with a low NPY receptor level (Y5R^low^) showed glycolysis signatures similar to those found in primary tumors (Fig. 4A). These results suggest the possibility that low BMI-induced neuronal NPY promotes metabolic reprogramming of tumor cells via tumor cell-specific Y5R. Therefore, we measured fatty acid oxidation (FAO) driven oxygen consumption rate (OCR) and glycolytic rate (ECAR) in control (ScrKO) and Y5rKO cancer cells in the presence or absence of recombinant NPY treatment (100 nM). We found a significant upregulation of FAO-driven OCR relative to glycolysis in NPY-treated control cells compared to untreated or Y5rKO cancer cells (Fig. 4B). We then examined whether NPY-induced fatty acid metabolism also exhibits an increased accumulation of lipids in the tumor cells. Using Oil-red O, a lipophilic dye that labels neutral lipids, we found a marked increase in the staining of lipid droplets in NPY-treated control cells compared to untreated or Y5rKO cancer cells (Fig. 4C, and Supplementary Fig. 5B). This result suggests that NPY promotes de novo lipogenesis in cancer cells, thereby enhancing tumor cell proliferation and survival. We therefore evaluated the expression level of SREBP2, a lipogenic regulator^66^ in the presence or absence of recombinant NPY protein in conjunction with Y5R^high^-specific pathways (PPAR, JAK/STAT3, PI3K) that were found to be enriched in lung cancer brain metastatic clinical samples (Fig. 3, panel E). Interestingly, among all, only the active SREBP2 level was found to be upregulated in NPY-treated control cells, in contrast to untreated or Y5rKO cancer cells as well as in NPY-treated parental lung cancer cells (Fig. 4D-E). This upregulation is positively correlated with ERK5 along with SREBP2, FASN and CPT1 in brain metastatic tumor tissue (*n* = 19)^67^ (Fig. 4F). Similar results were observed in TCGA lung cancer database (Supplementary Fig. 5C) and validated in control cells (ScrKO) treated with or without recombinant NPY protein (100 nM), in the presence or absence of the ERK5 inhibitor (100 nM), compared to untreated or Y5rKO cancer cells (Fig. 4G-H), providing further evidence for the regulatory role of NPY in modulating the ERK5/SREBP2 signaling pathway. Additionally, by querying an existing RNA-seq. dataset of lung cancer cells cultured with or without glucose (GSE199089)^68^, we found differential expression of genes controlling the fatty acid and cholesterol synthesis pathways in glucose-starved lung cancer cells (mimicking a low BMI condition) compared to cells exposed to glucose alone (Supplementary Fig. 5D). Next, to elucidate the molecular mechanism underlying NPY-mediated Y5R upregulation, we explored potential regulatory factors involved in the Y5R gene. Our analysis identified a putative SREBP2 transcription factor binding site within the 5’ region of Y5R (-515 relative to TSS), suggesting that SREBP2 may play a key role in mediating this effect. Furthermore, our clinical data from lung cancer brain metastatic patients showed a significant positive correlation between Y5R and ERK5 expression, as well as between ERK5 and SREBP2. Therefore, to validate these findings, we ectopically expressed either ERK5 or SREBP2 in cancer cells, with or without recombinant NPY, in the presence or absence of ERK5 or SREBP inhibitors. We found that overexpression of either ERK5 or SREBP2 led to significant upregulation of Y5R expression compared to controls, while their specific inhibitors significantly reduced the expression of Y5R (Fig. 4I). To further validated these results, we examined cancer cell proliferation in the presence or absence of CPT1 inhibitor. We found that CPT1 inhibition significantly reduced proliferation compared to controls (Supplementary Fig. 5E). These findings, consistent with our Y5R knockout metabolomic data, support the notion that NPY-induced metabolic changes via the SREBP2/FASN/CPT1 axis contribute to the proliferative effects during the early stages of metastatic colonization in the brain.

### Reversing the low BMI phenotype or blocking Y5R suppresses brain metastasis in lung cancer

**Fig. 5:**
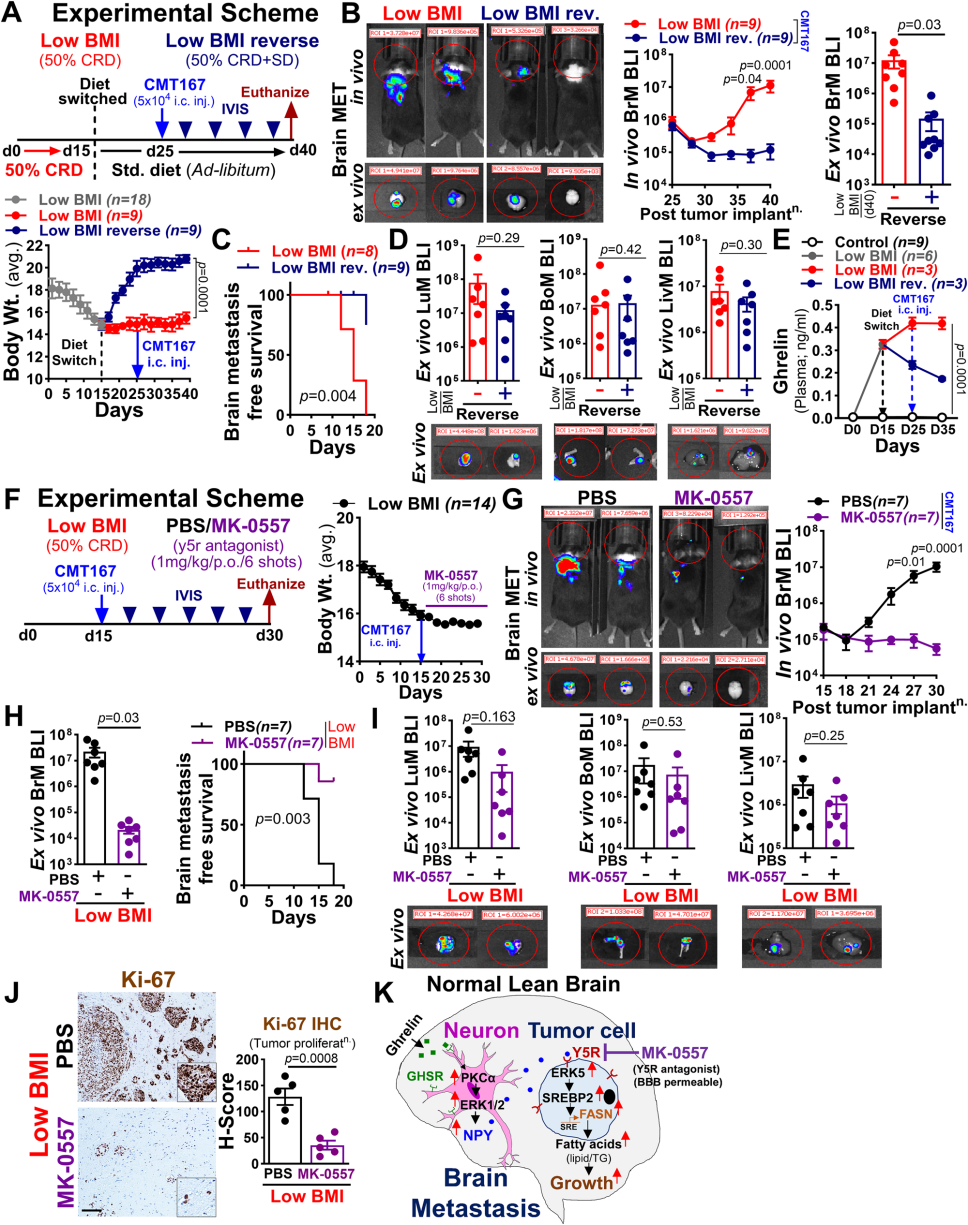
Low BMI reversibility or Y5R inhibition suppresses lung cancer brain metastasis. **A** Top: Schematic of in vivo metastasis assay. Female C57BL/6 mice (5-6 weeks) were fed with a 50% CRD (12-hr/dark phase; 15 days), then switched to standard diet (10 days). On day 25, mice received CMT167 cells injection (5×10^4^; i.c.). Bottom: Average body weight (two-way ANOVA with Tukey’s test). **B** Top left: Representative BLI images of brain metastasis at endpoint. Middle/Right: In vivo and ex vivo BLI quantification of brain metastasis in control (low BMI) and reversed low BMI mice (*n* = *9/*group) [two-way ANOVA with Tukey’s test (in vivo); unpaired two-tailed t-test (ex vivo)]. **C** Kaplan–Meier analysis of brain metastasis-free survival in control (*n* = *8*) and reversed low BMI mice (*n* = *9*) [log-rank (Mantel–Cox test)]. **D** Representative ex vivo BLI images and quantification of extracranial metastasis in control and reversed low BMI mice (*n* = *7/*group; unpaired two-tailed t-test). **E** Serum ghrelin level in control (*n* = *9*) and low BMI mice (*n* = *6*) at indicated time points before/after diet switching (two-way ANOVA with Tukey’s test). **F** Left: Schematic of in vivo metastasis assay. Female C57BL/6 mice (5-6 weeks) were fed with a 50% CRD (12-hr/dark phase; 15 days) before CMT167 cells injection (5 × 10^4^; i.c.). Mice were treated with PBS or MK-0557 (1 mg/kg/p.o./6-shots, *n* = *7*/group). Right: Average body weight. **G** Left: Representative BLI images of brain metastasis at endpoint. Right: In vivo BLI quantification of brain metastasis in PBS or MK-0557 treated mice (*n* = *7*/group; two-way ANOVA with Tukey’s test). **H** Left: Ex vivo BLI quantification of brain metastasis in PBS or MK-0557 treated mice (*n* = *7*/group; unpaired two-tailed t-test). Right: Kaplan–Meier analysis of brain metastasis-free survival in PBS or MK-0557 treated mice [*n* = *7/*group; log-rank (Mantel–Cox test)]. **I** Representative ex vivo BLI images and quantification of extracranial metastasis in PBS or MK-0557 treated mice (*n* = *7/*group; unpaired two-tailed t-test). **J** Left: Representative Ki-67 + IHC in metastatic brain tissue from PBS or MK-0557 treated mice [*n* = *5/*group; Scale bar: 100 µm (Ki-67), insert (2x Zoom)]. Right: Dot plot showing Ki-67 quantification by H-score (unpaired two-tailed t-test). **K** Mechanism of low BMI-induced brain metastasis via neuronal NPY/Y5R signaling. All data are mean ± S.E.M. Source data are provided as a Source Data file.

Because low BMI plays a significant role in the progression of brain metastasis in lung cancer, we examined whether reversing the low-BMI phenotype upon resuming a normal diet, either before or after cancer cell inoculation, would impact brain metastasis. We took a weight-loss and weight-regain diet intervention approach in a brain metastasis model by intracardially injecting CMT167 mouse lung cancer cells into syngeneic C57BL/6 mice to assess brain metastasis progression (Fig. 5A, and Supplementary Fig. 6A). Notably, we found that reversing the low BMI phenotype resulted in a significant increase in body weight in the low-BMI mice along with a significant decrease in brain metastatic burden by more than 20-fold compared to the control group that was maintained on a low-BMI condition (Fig. 5B, and Supplementary Fig. 6B). This led to significantly enhanced brain metastasis-free survival compared to the control group (Fig. 5C, and Supplementary Fig. 6B). Furthermore, mice exposed to the weight-regain intervention regimen did not show any significant decrease in extracranial metastatic sites (lung, bone, and liver) compared to brain metastasis (Fig. 5D, and Supplementary Fig. 6C), consistent with our clinical observation. To further validate our results, we examined the circulating levels of plasma ghrelin during the low BMI reversal at different time points throughout the course of brain metastasis progression. We found a significant decrease in plasma ghrelin levels in the mice that were subjected to weight-regain intervention on day 25 compared to the low-BMI control mice from day 15 to day 25. We also found a persistent decrease in the plasma ghrelin levels within the weight-regain mice post-cancer cell implantation (day 35), in contrast to low-BMI control mice, which showed a significant increase in plasma ghrelin levels during metastatic outgrowth (Fig. 5E), suggesting that low BMI is a severe but modifiable risk factor for brain metastasis in lung cancer. The aforementioned results indicate that ghrelin-mediated low-BMI activates neuronal NPY, which supports Y5R^high^ cancer cells in metastasis to the brain, providing direct evidence for the crucial role of the NPY/Y5R axis in brain metastasis. Therefore, identifying an interventional agent that specifically blocks NPY’s effect on tumor cells would be a promising therapeutic approach to treat lung cancer brain metastasis. Accordingly, we focused on the FDA-approved drug, MK-0557, a potent, highly selective, orally active, blood-brain barrier (BBB) permeable Y5R antagonist that underwent a clinical trial aiming to treat obesity but did not induce clinically meaningful weight loss^69,70^. We, therefore, tested the in vivo efficacy of MK-0557 (Y5R antagonist) on brain metastasis by implanting CMT167 lung cancer cells into syngeneic C57BL/6 mice followed by oral administration of MK-0557 (1 mg/kg; 6 shots till endpoint) under low-BMI condition (Fig. 5F). We found that MK-0557 significantly decreased low-BMI induced brain metastasis and thereby increased brain metastasis-free survival without any change in body weight (Fig. 5G–I). Furthermore, MK-0557 treatment did not induce any notable toxicity or weight loss in the treated mice (Supplementary Fig. 6D-E) and only showed effects on cancer cell viability and growth at higher doses compared to Y5R-KO cancer cells (Supplementary Fig. 6F). Importantly, ghrelin pre-treated neuron-conditioned media failed to rescue cancer cell proliferation under MK-0557 treatment suggesting inhibition of the NPY-specific growth-promoting effect on tumor cells (Supplementary Fig. 6G). We also examined the number of lipid droplets in brain metastatic tumor lesions from control and MK-0557 treated low-BMI mice by LipidSpot staining. We found that MK-0557 treatment significantly reduced the number of Ki-67 positive tumor cells as well as lipid droplets in brain metastatic tumor lesions of treated mice compared to the control mice (Fig. 5J, Supplementary Fig. 6H). Overall, these results provide a rationale for advocating the maintenance of normal body weight and exploring the blockade of NPY/Y5R interactions (Supplementary Fig. 7A–B). This could help determine its strength as a promising treatment approach for potential clinical intervention in treating brain metastasis in lung cancer patients based on BMI status. In summary, we propose the underlying mechanism by which, under the influence of ghrelin, low BMI induces neuronal activation and secretion of NPY via GHSR/PKCα/ERK axis, which binds to Y5R on tumor cells, triggering a metabolic switch from glucose to fatty acids, thereby promoting brain metastatic progression (Fig.5K).

## Discussion

A large body of epidemiological data has demonstrated a strong association between high BMI and an increased risk of cancer mortality across various cancers^71–74^. On the contrary, a negative correlation has been observed between high BMI and the progression or mortality of lung cancer^75–77^. Furthermore, meta-analyses conducted over the past decade using large cohort databases have identified the existence of the “obesity paradox” in advanced lung cancer compared to other cancers regarding the relationship between BMI and incidence^78–81^. However, the reason why high BMI is associated with a lower incidence of lung cancers and lower cancer-specific mortality in comparison to other cancers remains unclear. This raises the intriguing question of whether low BMI serves as a surrogate for cachexia, particularly in advanced lung cancer, which has a reported incidence of 40-50%, and is associated with poor overall survival^82^. BMI alterations are often attributed to an imbalance between energy intake and total energy expenditure (TEE)^83^, which may increase energy metabolism. This association could stem from patients’ failure to sufficiently increase their energy intake, leading to altered body composition that contributes to cachexia development. This trend is evident in a recent metastatic lung cancer clinical trial *(NCT0314)*^84^ and among healthy underweight adults^85^. These observations suggest that cancer cachexia may not be a causative factor but rather a consequence of low body weight (anorexia) due to hypermetabolism in metastatic lung cancer as recently reported^86^. However, the paradoxical prognostic and predictive relationships between low and high BMI in lung cancer patients in the metastatic setting have not been well understood. Interestingly, recent clinico-epidemiological studies have shown a positive correlation between low BMI and a 2-fold increased risk of brain metastasis in lung cancer patients as compared to those with a high BMI or other cancer types, which is further correlated with poor overall survival^9–13^. Nonetheless, the nuanced relationship between low BMI and brain metastasis warrants further research, particularly in understanding the underlying molecular mechanisms, with potential implications for the management and prognosis of metastatic lung cancer patients with low BMI. We demonstrated that low BMI serves as a robust predictor of adverse outcomes, suggesting a potential association between nutritional status, metabolic alterations, and reduced survival outcomes, with an unfavorable prognosis for metastatic lung cancer, which profoundly impacts the patient’s quality of life. Overall, our findings offer potential implications for early identification and intervention in patients with low BMI. This underscores the necessity for tailored interventions, including BMI monitoring upon initial admission, effective nutritional support, and dietary modification to address metabolic dysregulation, which could lead to improved treatment outcomes and prognosis in patients with metastatic lung cancer and low BMI.

Ghrelin is an orexigenic peptide primarily secreted by the stomach, playing a crucial role in regulating appetite, body weight, energy expenditure, and growth hormone secretion^34,35,38,87^. This peptide modulates neuronal activity by traversing the blood-brain barrier (BBB) and blood-cerebrospinal fluid barrier (BCB)^33,37,39,88^. Moreover, ghrelin directly interacts with neurons via multiple mechanisms through its receptor, GHSR (a G-protein-coupled secretagogue receptor)^36^, and promotes neurogenesis in diet-restricted mice^89,90^. Conversely, ghrelin exhibits an inhibitory effect on other brain cell types expressing GHSR, such as astroglia and microglia, to protect neurons from the adverse effects of pro-inflammatory factors released during neurodegenerative disease or injury processes^91–94^. In addition to its neuroprotective role, ghrelin also plays a role in synapse formation and the generation of long-term potentiation, contributing to enhanced spatial learning and memory^95^, as well as additional physiological functions such as psychological stress, mood, anxiety^96^, depression^97^, and aging^98^. Interestingly, increased serum ghrelin levels have been previously reported to induce body weight loss (anorexia) but not muscle mass loss (cachexia) in lung cancer patients^99,100^. Additionally, prior studies have indicated elevated circulating ghrelin levels in lean and healthy individuals undergoing fasting, calorie restriction regimes, or exercise regimens, as opposed to individuals with normal weight, overweight, or non-exercising status. This reflects a negative feedback mechanism to maintain energy homeostasis in non-conducive environments^40,42–44,101–103^. Notably, we found a significant inverse correlation between high plasma ghrelin levels and low BMI in cancer-free subjects and in mice with brain metastasis, with its levels significantly decreased in response to a weight-loss and weight-regain diet intervention approach in vivo, leading to increased brain-metastasis free survival. These findings suggest that circulating ghrelin levels could serve as a promising prognostic biomarker for predicting an increased risk of brain metastasis in lung cancer patients with low BMI. Moreover, existing findings demonstrate the tumor-suppressive role of ghrelin in various cancers^104–106^, consistent with our data. Furthermore, the specific activation of neurons by ghrelin compared to other brain cell types under low BMI conditions implicates its functional role in supporting neuron survival during metabolic adaptation to calorie restriction, in agreement with previous studies demonstrating GHSR receptor-mediated enhanced neuronal activity in energy-deficit states^107–110^. Taken together, these observations highlight the intricate role of ghrelin in mediating the low-BMI phenotype and suggest a relationship between GHSR-activated neurons and the development of metastatic disease in lung cancer patients with low BMI. Interestingly, accumulating evidence suggests that metformin, the anti-diabetic drug, directly inhibits ghrelin production and induces weight loss in both diabetic and non-diabetic individuals^111–115^. However, the impact of inherently lower ghrelin levels and the use of metformin in these individuals, particularly in the context of brain metastasis, is not yet fully understood. Notably, recent data indicate a significantly lower incidence of brain metastases in diabetic melanoma patients treated with metformin^116,117^. Furthermore, meta-analyses^118–121^ have shown that diabetic patients receiving metformin exhibit a reduced risk of lung cancer compared to non-diabetic individuals. These findings suggest that metformin may play a confounding role in modulating lung cancer progression, highlighting the need for further mechanistic investigations.

Our study unveiled a significant increase in neuronal NPY expression and secretion in the brains of low BMI cancer-free subjects and tumor-free mice, as well as in ghrelin-pretreated primary neurons. Similarly, previous findings have shown a ∼2-fold increase in brain NPY levels in ghrelin-activated neurons under fasting, in underweight anorexia patients, and during food-deprived conditions^122–125^, which is significantly attenuated by pharmacological inhibition of ghrelin-mediated MAPK1 signaling in neurons^48,126^, highlighting ghrelin’s specific role in neuron activation and NPY secretion. Furthermore, our study demonstrated a significant increase in tumor growth upon NPY treatment, along with an increased brain metastatic burden in a low BMI setting, indicating its distinct role in lung cancer progression beyond regulating feeding behavior and energy homeostasis^127,128^. This was consistent with previous studies implicating dysfunction of the NPY system in various inflammatory pathologies, including cardiovascular disease, and neuroendocrine tumors through distinct signaling pathways via their receptors (Y1R, Y5R)^57,58,129–131^. Notably, we found significantly activated NPY receptor activity with substantially higher Y5R expression in matched brain metastatic tumors in contrast to primary tumors, or other NPY receptors or metastatic breast and melanoma cancers and this was significantly correlated with low BMI, resulting in lower brain metastasis-free survival in a Y5R/MAPK-dependent mechanism, indicating that MAPK1 serves as a key oncogenic driver contributing to the downstream effect of NPY in cancer cells. Nevertheless, our findings strongly suggest a critical role of the Y5R in mediating low BMI-induced neuronal NPY effects via the MAPK pathway in lung cancer progression and offer a potential therapeutic avenue to treat MAPK-driven cancers using ERK inhibitors, particularly in metastatic lung cancer patients with low BMI.

Recent evidence suggests that metabolic plasticity is a crucial determinant of tumor metastasis^64^. Differences in nutrient availability in brain tissue relative to other tissues can necessitate metabolic adaptation or competition by cancer cells for growth in the brain^65,132,133^. Furthermore, NPY has been shown to play a significant role in regulating metabolic homeostasis and energy balance, as observed in condition such as obesity, hypertension, and atherosclerosis^134^. In this regard, we identified enrichment of activated signatures specific to fatty acid (FA) metabolism, biosynthesis, and oxidation in brain metastasis compared to primary tumors. Importantly, fatty acid metabolism has been shown to play a pro-tumoral role in cancer cells^135,136^. Although the underlying molecular mechanism(s) of this metabolic rewiring remains unclear, emerging evidence suggests that FA metabolism accelerates the incidence of brain tumor or metastasis, as both glycolysis and FAO pathways are essential for tumor metabolism, dictating tumor aggressiveness^65,137–141^. Such bioenergetic flexibility appears to fit well with the increased capacity of tumors for metastasis, in which fatty acids serve as an alternative critical energy resource to meet the high-fuel consumption of aggressively growing cancer cells. Intriguingly, our study revealed significantly upregulated FA metabolism, mainly FA-oxidation (FAO), in brain metastatic tumors under low BMI conditions, which was further enhanced upon NPY treatment in a Y5R-dependent manner. These findings are consistent with existing literature indicating tumor cells’ dependency on elevated fatty acid metabolism for their survival in a non-conducive environment in metastatic brain tumors^65,138,140–142^. Moreover, fasting and calorie restriction regimens have been shown to increase FAO rates along with FA synthesis to maintain homeostasis^143,144^, supporting our findings from clinical and pre-clinical models of brain metastasis with low BMI. Additionally, we observed elevated intracellular lipid droplets in Y5R^high^-tumor cells in correlation with increased levels of ERK, SREBP2, FASN and CPT1 in clinical samples of lung cancer brain metastasis and in patient-derived brain-metastatic xenografts. This effect was attenuated in the presence of an ERK5 inhibitor, indicating NPY-mediated active de novo lipogenesis in Y5R^high^-tumor cells through an ERK5/SREBP2-dependent mechanism, consistent with existing literature highlighting Y5R involvement in mediating lipogenesis^145^. Collectively, our results suggest that the energy derived from FAO contributes to supporting tumor growth during brain colonization under a low-BMI condition through the NPY/Y5R signaling axis.

By utilizing the highly selective, orally active, and blood-brain barrier (BBB) permeable Y5R antagonist, MK-0557^69,70^, we found that a low dose (1 mg/kg) effectively abrogated low BMI-induced brain metastasis and significantly increased brain-metastasis free survival in vivo by blocking NPY/Y5R axis. Importantly, MK-0557 demonstrated this efficacy without affecting overall health or body weight in low BMI mice and intriguingly suppressed cancer cell viability and growth only at higher doses. However, its impact on body weight in normal mice remains poorly understood, as most studies on Y5R inhibition have primarily focused on obese or high-fat diet models^69,146,147^, necessitating further investigation. Notably, MK-0557 treatment resulted in a significant reduction in intracellular lipid droplet formation within brain metastatic lesions. Considering its selective in vivo inhibitory effect on the Y5R, along with its relatively low toxicity and BBB permeability, MK-0557 emerges as a potential therapeutic agent to suppress the onset of brain metastasis, particularly in lung cancer patients with low BMI. Our study provides insights into the mechanisms underlying low BMI-associated alterations in the interplay between tumor cells and brain cells. Specifically, we have elucidated the role of neuronal NPY in driving brain metastasis and highlighted its functional significance in regulating cancer cell survival and growth by reprogramming their metabolism. Moreover, the identification of elevated plasma ghrelin levels in individuals with low BMI holds significant clinical importance, particularly as a reliable prognostic marker for patients with lung cancer and low BMI. Additionally, the recognition of tumor metabolic dependency on fatty acid metabolism unveils a promising pharmacological avenue to effectively suppress NPY-activated cancers in the brain. Furthermore, the exploration of prospective dietary interventions and the utilization of the Y5R antagonist presents promising clinical translational applications for the treatment of lung cancer brain metastasis in patients with low BMI.

## Methods

### Human samples

Formalin-fixed paraffin-embedded (FFPE) tumor tissues from lung cancer brain metastatic patients with a BMI history were obtained from the Tumor Tissue and Pathology Shared Resource at Wake Forest Baptist Comprehensive Cancer Center. All samples were deidentified and collected in accordance with the Wake Forest School of Medicine IRB-approved protocol (IRB00107587). Informed consent was obtained from all participants. Demographic details, including BMI, age, gender, race, and smoking status were provided in the source data file.

### Cell culture and reagents

Human lung cancer brain-tropic cell lines H2030BrM and PC9BrM (derived from in vivo selection of H2030, PC9 cell line harboring the *Kras* mutation)^148^ were a kind gift from Dr. Massague (Memorial Sloan-Kettering Cancer Center). Mouse lung carcinoma cell lines, LL/2 and CMT167, were purchased from American Type Culture Collection (ATCC) and MilliporeSigma. iPSC-derived GABAergic human primary neurons were purchased from BrainXell and maintained in neurobasal medium (ThermoFisher), supplemented with GlutaMAX (ThermoFisher), pen/strep (ThermoFisher), B-27 supplement (ThermoFisher), N-2 supplement (ThermoFisher), and BDNF (10 ng/mL; ThermoFisher). E6/E7/hTERT immortalized human astrocyte (UC1) were a kind gift from Dr Russell Piper (University of California-San Francisco) and were cultured in DMEM with 10% FBS. Human microglial cell line HMC3 was purchased from ATCC, authenticated by ATCC and cultured in DMEM/F12 medium supplemented with 5% FBS. All cells were cultured in their respective medium supplemented with 10% FBS, streptomycin (100 mg/ml), and penicillin (100 units/ml). All cells were grown at 37 °C in a 5% CO_2_ atmosphere and were routinely tested for the absence of Mycoplasma.

### Animal experiments

All animal experiments were done in accordance with the U.S. National Institutes of Health Animal Protection Guidelines, and the protocol was approved by the Wake Forest Baptist Health Institutional Animal Care and Use Committee. All mice (C57BL/6; female, 5-6 week) were housed individually and were maintained in a specific pathogen free unit on a 12:12 light: dark cycle with either *ad-libitum* standard diet (5P00 - Prolab® RMH 3000 from LabDiet) or 50% calorie restriction diet (D19021107 from ResearchDiets) with time-restricted feeding and access to water. The animal rooms were provided with 100% fresh, HEPA filtered air at 10-15 air changes per hour. Room temperatures were controlled by reheat units within each room, and were maintained within the range of 70 °F ± 2° F. The humidity levels were controlled and maintained between 30-70%. Sample size was calculated based on previous experiments^149,150^. Using power analysis, the anticipated means and standard error of means were included to determine the sample size that was expected to yield a power of approximately 80 percent using a *p*-value of 0.05. All mice were age- and sex-matched and randomized into different experimental groups.

To examine the effect of BMI, we established a low-BMI mouse model (Supplementary Table 1) through a regimen of 50% calorie restriction combined with time-restricted feeding (mimicking human low BMI condition) and examined its role in experimental metastatic models by implanting luciferase-labeled CMT167 and LL2 mouse lung cancer cells in syngeneic C57BL/6 mice via intracardiac injection (i.c.) followed by monitoring the tumor metastatic burden by bioluminescence imaging (BLI). All mice were randomized before the experiment, blindly selected before injection. To confirm successful injection, whole-body bioluminescence imaging (BLI) was immediately performed using the IVIS imaging system. Tumor growth reaching a radiance of 10^8^ photons/sec/cm^2^/sr was considered the humane endpoint. Tumor progression was monitored twice weekly using BLI. At the endpoint, mice were sacrificed, and the whole brain, lung, liver and bones were removed, and the presence of metastatic lesions on these organs was evaluated by ex vivo BLI imaging. The BLI images were analyzed using the Xenogen IVIS imaging system (Caliper Life Science).

### Bioluminescence imaging (BLI)

For in vivo monitoring of tumor growth, bioluminescence imaging was performed using an IVIS imaging system (Xenogen). Animals were placed under 1-4% isoflurane anesthesia and injected with luciferin substrate. Images were then acquired and analyzed using the IVIS imaging system (Xenogen). Baseline bioluminescence was used to randomize animals by a blinded investigator.

### Histological staining

Hematoxylin and eosin (H&E) staining was performed on formalin-fixed paraffin-embedded (FFPE) tumor tissues using standard protocols. Briefly, tissue was fixed in 10% formalin overnight, dehydrated, embedded in paraffin, and sectioned at 4–8 μm thicknesses using a microtome and processed for H&E staining. For immunohistochemistry (IHC), paraffin-embedded sections were de-paraffinized, rehydrated, and heated at 100 °C for 20 min in Tris-buffered saline (pH 9.0) for antigen exposure. The sections were treated with 3% H2O2 to block endogenous peroxidase activity, followed by incubation in 5% BSA solution for 30 min. The slides were then incubated with primary antibody, Ki-67 (Cell Signaling Technology, clone 24E10, #3195, 1:200) for 16 h at 4 °C. After washing in PBS/0.1% Tween-20, the sections were incubated with anti-rabbit secondary antibody (Bio-Rad, #1706515, 1:500). The sections were washed extensively, treated with DAB substrate chromogen solution, and counterstained with hematoxylin. For the negative control, the rabbit IgG isotype control (Invitrogen, 02-6102, 1:250) was used in place of primary antibody. The expression of the protein of interest was evaluated by H scoring, which was calculated as the sum of the frequency and intensity (0: none; 1: weak; 2: moderate; 3: strong). The final score = 1 × (% of 1 + cells) + 2 × (% of 2 + cells) + 3 × (% of 3 + cells). Image quantification was done using ImageJ software. IHC scores were determined based on concordance between two independent reviewers.

### Quantitative real-time PCR (RT-qPCR)

Total RNAs were extracted from indicated cells or FFPE sections using either the Direct-zol RNA kit (Zymo research) or the RNeasy FFPE kit (Qiagen), according to the manufacturer’s instructions. The concentration of RNA was quantified using a NanoDrop 2000 UV-spectrophotometer (Thermo Scientific) and reverse transcribed into complementary DNA (cDNA) using the Reverse Transcription Supermix kit (Bio-Rad, USA). The cDNA was then amplified using a pair of forward and reverse primers for the genes listed in Supplementary Table 3. Results were normalized to the housekeeping gene β-Actin. The thermal cycling conditions consisted of an initial denaturation step at 95 °C for 1 min., followed by 35 PCR cycles with the following profile: 94 °C for 30 s, 62 °C for 30 s, and 72 °C for 30 s, using the Bio-Rad CFX connect.

### Western blotting

Western blotting was performed using standard method. Briefly, total protein from cells was extracted using radioimmunoprecipitation assay buffer with Halt protease inhibitor cocktail (Thermo Fisher Scientific). Plasma membrane protein was extracted by the Plasma Membrane Protein Extraction Kit (Abcam). Protein concentration was measured using a protein assay (Bio-Rad). Equal amounts of protein were resolved by 10% SDS–polyacrylamide gel electrophoresis, followed by electro-transfer to nitrocellulose membranes. Primary antibodies used included PKCα (1:1000, Cell Signaling, #59754 T), pPKCα (1:1000, Cell Signaling, #44-962 G), ERK5 (1:1000, Cell Signaling, #D23E9), pERK5 (1:10000, ThermoFisher, #44-612 G), FoxO1 (1:1000, Cell Signaling, #2880 T), AMPKα (1:1000, cell Signaling, #2532S), pAMPKα (1:1000, cell Signaling, #2535 T), SirT1 (1:1000, cell Signaling, #8469 T), p53 (1:1000, cell Signaling, #2527 T), pCREB (1:1000, Cell Signaling, #9198 T), pSTAT3 (1:1000, Cell Signaling, #9145S), PPARγ (1:1000, Cell Signaling, #2443), FASN (1:1000, cell Signaling, #3180 T), SREBP2 (1:500, Novus Biologicals, #NB100-74543), pSREBP2 (1:500, ThermoFisher, #PA5-106042), NPY (1:500, Cell Signaling, #11976 T), NPY5R (1:500, ThermoFisher, #PA5-106850), BSX (1:100, MyBioSource.com, #MBS9609602) and GAPDH (1:10000, Cell Signaling, #5174 T). HRP-conjugated anti-mouse IgG (1:5000, Cell Signaling, #7076) or anti-rabbit IgG (1:5000, Bio-Rad, #1706515) were used as secondary antibodies. Bound antibodies were detected using an enhanced chemiluminescence detection kit (GE Healthcare Lifescience). Band intensities were measured using an Amersham Imager 600 and analyzed using ImageJ (version 1.53c http://rsb.info.nih.gov/ij/).

### ELISA

Ghrelin and NPY levels in plasma and conditioned media were measured using the mouse Ghrelin and human NPY ELISA Kits according to the manufacturer’s instructions (MyBioSource; RayBiotech). A standard curve was generated using known concentrations of ghrelin and NPY provided by the kit, and the concentration of each sample was interpolated from it. All concentration was determined at a 450 nm wavelength using an EMax Plus microplate reader (Molecular Devices, CA).

### Generation of conditioned medium

Conditioned medium (CM) was generated from cultured human microglia, astrocytes lines (HMC3, UC1), followed by centrifuge at 300 g for 10 min to remove cells and debris, then stored at -80°C. CM from human primary neurons was prepared by seeding 2x10^6^ cells with or without recombinant ghrelin (0.01-0.25 nM) or recombinant neuropeptide (10-100 nM) in culture plates coated with poly-L-lysine (Sigma). After overnight incubation, cells were washed with PBS and further incubated in serum-free neuron-specific media for 24 h. The harvested CM was then centrifuged to remove cell debris (300 g/10 min) and stored at -80°C for further analysis.

### MTS assay

The 3-(4,5-dimethylthiazol-2-yl)-5-(3-carboxymethoxyphenyl)-2-(4-sulfophenyl)-2H-tetrazolium (MTS Promega) assay was used to determine cells viability with indicated drugs in a time- and dose-dependent manner. Briefly, cancer cells were seeded into 96-well plates and incubated overnight at 37 °C. The cells were then treated with indicated recombinant proteins or brain cell-specific CM in dose- and time-dependent manner. Thereafter, MTS solution was added to each well, and the plates were incubated for 4 h at 37 °C. The media was then removed, and 150 µl DMSO was added to dissolve the formazan crystals. Absorbance was measured at 490 nm using the EMax Plus microplate reader (Molecular Devices, CA). Four to five replicate wells were included in each analysis, and at least three independent experiments were conducted.

### BrdU incorporation assay

Cancer cells were treated with or without recombinant NPY or ghrelin with the desired concentration as indicated in the figure. BrdU incorporation assay was performed using FITC BrdU Flow kit (BD Biosciences) according to the manufacturer’s instructions. Briefly, cells were pulsed with 10 μM BrdU and stained with anti-BrdU antibody following DNA digestion. Cells were then stained with nuclear stain 7-AAD, and data was recorded by flow cytometry using the FACS Canto II flow cytometer and FACSDiva software (BD Biosciences) and analyzed by FlowJO. The gating strategy used for flow cytometric analysis of BrdU incorporation is provided in Supplementary fig. 8.

### Colony formation assay

Cells were treated with or without conditioned media (CM) derived from indicated brain cell types, or recombinant NPY, or ghrelin (to control media) and allowed to grow for 7 days. The media were replenished every 3^rd^ day. On day 7, the colonies were fixed with ethanol for 30 min, stained with Crystal Violet (CV) for 20 min., and then the number of colonies was counted manually.

### Oil red O staining

Control and treated cells were washed and fixed in 4% paraformaldehyde at room temperature for 1 h. After washing three times with 60% isopropanol, the cells were stained with 60% filtered Oil Red O working solution (vol/vol in distilled water) prepared from Oil red O stock solution (#0843, ScienceCell Research) at room temperature for 15 min. The cells were then washed with ddH2O before imaging. To quantify lipid accumulation, Oil Red O-stained lipids were eluted in 100% isopropanol, and then the optical density (OD) was measured at 500 nm.

### Lipid droplet staining

For the lipid assay, 610 dye (#70069, Biotium) was used. Cells were incubated directly with LipidSpot^TM^ 610 dye at a 1:1000 dilution for 1 h, protected from light in the incubator. Images were captured using the Keyence fluorescence microscope (BZ-X710).

### Fatty Acid β-Oxidation (FAO) driven Oxygen Consumption Rate (OCR)

FAO measurement was done by following the manufacturer’s instruction of Fatty Acid Oxidation Assay Kit from (Abcam, ab222944). Briefly, control or y5r-KO CMT167 cancer cells were seeded per well in 96-well plates and cultured overnight. Then the cells were washed twice with 100 μl prewarmed FA-Free medium followed by adding 90 μl pre-warmed FA-Measurement medium. The wells without cells were used as signal control. A total of 85 μl of FA-Free Measurement medium was added to the wells, and 5 μl of BSA control were included as the FA-free control. All wells except the blank control had 10 μl Extracellular O_2_ Consumption Reagent added. The FAO activator FCCP (0.625 μM) and inhibitor Etomoxir (40 μM) with or without FAO-conjugate (Oleate-BSA, 2:1) were added as positive and negative controls. Then the wells were sealed with 100 μl pre-warmed mineral oil, and the FAO was measured using the condition as Extracellular Oxygen Consumption by measuring fluorescence using Tecan microplate reader.

### Extracellular acidification rate (ECAR)

For ECAR assay (Abcam, ab197244), control or y5r-KO CMT167 cancer cells were cultured for 48 h, and culture media was changed to the glycolysis buffer containing probe and the rate of glycolysis was determined using Glycolysis Assay Kit (Abcam, US) following manufacturers recommended using Tecan microplate reader.

### CRISPR/Cas9 based gene knockout (KO)

Npy5r knockout was performed using the CRISPR/Cas9 system. Briefly, CMT167 cancer cells were transfected with Cas9 protein V2 (ThermoFisher) and two guide RNA sequence (sgRNA: GUAGUCCUCCCAGGCAGGG; UACUGCUGCCAGUCAGAACA) targeting the y5R sequence. Single-cell clones were isolated through limiting dilution following Synthego’s protocol, and successful knockout was confirmed by western blot analysis.

### Plasmids

Expression plasmids for mouse Srebp2 and pCI-HA-Erk5-FL were obtained from Addgene. Each plasmid (1 µg) was independently transfected into LL2 lung cancer cells via either by lentiviral method or Lipofectamine 3000 (Invitrogen). Following transfection, cells were treated with or without recombinant Npy (100 nM) in presence or absence of Erk5 or Srebp inhibitors (Selleckhem). After 24 h, western blotting was conducted using the standard protocol described in western blotting section, employing respective primary and secondary antibodies.

### MK-0557 (NPY5R antagonist) dosing

MK-0557 was purchased from MedChemExpress (cat. no. HY-15411) and reconstituted to a concentration of 1 mg/ml MK-0557 according to the manufacturer’s instructions. Mice received 50–100 µl of vehicle or 1 mg/kg MK-0557 via oral gavage (p.o.) immediately after intracardiac injection with 5 × 10^4^ CMT167-luc cells for the indicated duration.

### RNA-sequencing data from published studies

RNA-sequencing data from lung cancer brain metastasis patient cohorts (GSE200563, GSE123902)^55^, BrM-xenograft (GSE115699)^151^, were analyzed by downloading data from their respective source file or utilizing supporting interactive web portals (http://bmxexplorer.gotdns.org/) and discovery platforms such as Rosalind (https://www.rosalind.bio/). The data were further processed for Gene Set Enrichment Analysis (GSEA) to investigate oncogenic and metabolic pathways. For overall or organ-specific free survival (OS), samples were either stratified by high or low expression of the gene of interest, or by BMI status and were presented as Kaplan–Meier plots and tested for significance using log-rank tests as previously described^152^.

### Gene set enrichment analysis

GSEA was performed by generating the Gene MatriX file (.gmx) using published oncogenic signatures. The Gene Cluster Text file (.gct) was generated from the GEO database (GSE200563; GSE123902) either by separating primary and brain metastatic tumors or by stratifying brain metastatic tumors based on high or low expression of the gene of interest (Y5R). The Categorical class file (.cls) was generated based on the neuropeptide receptor activation scores and we used GPL21697, GPL16791 as the chip platform.

### ALT/AST Activity Assay

The ALT (alanine aminotransferase) activity was detected using ALT Activity Assay kit (Sigma-Aldrich, USA) according to the product information sheet. Briefly, 100 µL of the Master Reaction Mix was added to each of the standard, positive control, and test samples in each well of 96-well plates. After 2–3 min (T_initial_), take the initial measurement of absorbance at 570 nm (A_570_)_initial_. The plate was then incubated at 37 °C protected from light, and measurements were taken every 5 min until the highest value of the samples is greater than the highest value of the standards. The penultimate reading (T_final_) was the final measurement for calculating the enzyme activity (A_570_)_final_. To calculate the ALT activity, the background was corrected by subtracting the value obtained for the blank standard from all standard readings. The amount of pyruvate generated (B) between T_initial_ and T_final_ was calculated by plotting the ΔA_570_ = (A_570_)_final_ – (A_570_)_initial_ to the to the pyruvate standard curve. AST (aspartate aminotransferase activity was measured with AST Activity Assay kit (Sigma-Aldrich, USA) according to product information sheet. Briefly, 100 µL of the Reaction Mix was added to each of the well. After 2–3 min (T_initial_) incubation at 37 ˚C, initial measurement of absorbance at 450 nm (A_450_)_initial_ was taken. The measurements were then taken every 5 min until the value of the most active sample is greater than the value of the highest standard. The penultimate reading (T_final_) was the final measurement for calculating the enzyme activity (A_450_)_final_. To calculate the ALT activity, background was corrected by subtracting the value obtained for the blank standard from all standard readings. The amount of glutamate generated (B) between T_initial_ and T_final_ was calculated by plotting the ΔA_450_ = (A_570_)_final_ – (A_570_)_initial_ to the glutamate standard curve. Both ALT, AST activity was calculated as B × Sample Dilution Factor/ ((T_final_ – T_initial_) × Sample volume).

### Statistical analysis and reproducibility

The number of mice used in each experimental group was determined by power analysis and on the basis of prior experiences. All the statistical analysis was performed using the Prism 10.0 (GraphPad). Data are expressed as mean ± S.E.M. The *P* value was calculated using an unpaired Student’s t-test or ANOVA, with corresponding numbers (n) as indicated in the figures and their respective figure legends. Gene correlation analysis was calculated by linear regression analysis. Kaplan–Meier survival analysis was calculated using the log-rank (Mantel–Cox) test. Representative experiments were repeated independently at least three times with similar results. Significance between each group is represented as *P < 0.05, **P < 0.01, ***P < 0.001, ****P < 0.0001, unless otherwise indicated.

### Reporting summary

Further information on research design is available in the Nature Portfolio Reporting Summary linked to this article.

## Supplementary information

Supplementary Information

Reporting Summary

Transparent Peer Review file

## Source data

Source Data

## Data Availability

Previously published micro-array, RNA-seq. and proteomic data that were reanalyzed in this study are available in GEO under accession codes GSE200563, GSE123902, GSE48433, GSE14020, GSE199089, GSE50493 or at PRIDE - Proteomics Identification Database under accession codes PXD027259. Human protein atlas, GTEx database, Kaplan–Meier survival analysis, TCGA lung, Gene Ontology referenced during the study are available in a public repository from https://www.proteinatlas.org/; https://www.gtexportal.org/home/; http://kmplot.com/analysis/index.php?p=service; http://geneontology.org/https://lce.biohpc.swmed.edu/lungcancer/; website. Source data are provided as a Source Data file. Source data are provided with this paper.

## References

[1] Lamba, N., Wen, P. Y. & Aizer, A. A. Epidemiology of brain metastases and leptomeningeal disease. *Neuro Oncol.* **23**, 1447–1456 (2021). 33908612 10.1093/neuonc/noab101 PMC8408881

[2] Sacks, P. & Rahman, M. Epidemiology of brain metastases. *Neurosurg. Clin. N. Am.* **31**, 481–488 (2020). 32921345 10.1016/j.nec.2020.06.001

[3] Suh, J. H. et al. Current approaches to the management of brain metastases. *Nat. Rev. Clin. Oncol.* **17**, 279–299 (2020). 32080373 10.1038/s41571-019-0320-3

[4] Moss, N. S., Beal, K. & Tabar, V. Brain metastasis-a distinct oncologic disease best served by an integrated multidisciplinary team approach. *JAMA Oncol.* **8**, 1252–1254 (2022). 35862025 10.1001/jamaoncol.2022.1928 PMC9984120

[5] An, N. et al. Risk factors for brain metastases in patients with non-small-cell lung cancer. *Cancer Med* **7**, 6357–6364 (2018). 30411543 10.1002/cam4.1865 PMC6308070

[6] Barnholtz-Sloan, J. S. et al. A nomogram for individualized estimation of survival among patients with brain metastasis. *Neuro Oncol.* **14**, 910–918 (2012). 22544733 10.1093/neuonc/nos087 PMC3379797

[7] Sun, D. S. et al. A systematic review of risk factors for brain metastases and value of prophylactic cranial irradiation in non-small cell lung cancer. *Asian Pac. J. Cancer Prev.* **15**, 1233–1239 (2014). 24606446 10.7314/apjcp.2014.15.3.1233

[8] Waqar, S. N. et al. Brain metastases at presentation in patients with non-small cell lung cancer. *Am. J. Clin. Oncol.* **41**, 36–40 (2018). 26359696 10.1097/COC.0000000000000230 PMC4833709

[9] He, Y. et al. Association of underweight and weight loss with poor prognosis and poor therapy effectiveness in brain metastases: a retrospective study. *Front Nutr.* **9**, 851629 (2022). 35845778 10.3389/fnut.2022.851629 PMC9286517

[10] Lareida, A. et al. Underweight and weight loss are predictors of poor outcome in patients with brain metastasis. *J. Neurooncol* **145**, 339–347 (2019). 31571112 10.1007/s11060-019-03300-1

[11] Masel E. K. et al. Decreased body mass index is associated with impaired survival in lung cancer patients with brain metastases: a retrospective analysis of 624 patients. *Eur. J. Cancer Care (Engl)* **26**, 1–5 (2017). 10.1111/ecc.12707 28488812

[12] Nam, M. W., Kim, J. M., Cheong, J. H., Ryu, J. I. & Han, M. H. Paradoxical relationship between body mass index and bone mineral density in patients with non-small cell lung cancer with brain metastasis. *PLoS One* **14**, e0218825 (2019). 31226155 10.1371/journal.pone.0218825 PMC6588256

[13] Tsang, N. M. et al. Overweight and obesity predict better overall survival rates in cancer patients with distant metastases. *Cancer Med* **5**, 665–675 (2016). 26811258 10.1002/cam4.634 PMC4831285

[14] Banna, G. L. et al. Efficacy outcomes and prognostic factors from real-world patients with advanced non-small-cell lung cancer treated with first-line chemoimmunotherapy: the Spinnaker retrospective study. *Int Immunopharmacol.* **110**, 108985 (2022). 35777264 10.1016/j.intimp.2022.108985

[15] Cortellini A. et al. Baseline BMI and BMI variation during first line pembrolizumab in NSCLC patients with a PD-L1 expression >/= 50%: a multicenter study with external validation. *J. Immunother. Cancer* **8**, e001403 (2020). 10.1136/jitc-2020-001403 PMC7574933 33077515

[16] Cortellini A. et al. Prognostic effect of body mass index in patients with advanced NSCLC treated with chemoimmunotherapy combinations. *J. Immunother. Cancer* **10**, e004374 (2022). 10.1136/jitc-2021-004374 PMC8852707 35173031

[17] Crane, G. et al. Application of clinical trial inclusion criteria to clinical practice patients to quantify the burden of CNS metastases on health-related quality of life and healthcare resource use in patients with NSCLC. *Lung Cancer* **149**, 144–153 (2020). 33017726 10.1016/j.lungcan.2020.09.007

[18] Daly, L. E. et al. The impact of body composition parameters on ipilimumab toxicity and survival in patients with metastatic melanoma. *Br. J. Cancer* **116**, 310–317 (2017). 28072766 10.1038/bjc.2016.431 PMC5294486

[19] Dawood, S. et al. Incidence of brain metastases as a first site of recurrence among women with triple receptor-negative breast cancer. *Cancer* **118**, 4652–4659 (2012). 22359359 10.1002/cncr.27434 PMC3910255

[20] Di Filippo, Y. et al. Relevance of body mass index as a predictor of systemic therapy outcomes in metastatic melanoma: analysis of the MelBase French cohort data(☆). *Ann. Oncol.* **32**, 542–551 (2021). 33385520 10.1016/j.annonc.2020.12.012

[21] Lammers, S. W. M. et al. The prognostic impact of BMI in patients with HR+/HER2- advanced breast cancer: a study of the SONABRE registry. *Breast Cancer Res Treat.* **203**, 339–349 (2024). 37878148 10.1007/s10549-023-07108-6 PMC10787675

[22] Santoni M. et al. Body mass index in patients treated with cabozantinib for advanced renal cell carcinoma: a new prognostic factor? *Diagnostics (Basel)* **11**, 138 (2021). 10.3390/diagnostics11010138 PMC7831923 33477676

[23] Santoni, M. et al. Clinico-pathological features influencing the prognostic role of body mass index in patients with advanced renal cell carcinoma treated by immuno-oncology combinations (ARON-1). *Clin. Genitourin. Cancer* **21**, e309–e319 e301 (2023). 37062658 10.1016/j.clgc.2023.03.006

[24] Siringo M. et al. Evaluation of efficacy of ALK Inhibitors according to body mass index in ALK rearranged NSCLC patients-a retrospective observational study. *Cancers (Basel)* **15**, 3422 (2023). 10.3390/cancers15133422 PMC10340506 37444532

[25] Chen Y. M. et al. Body mass index, weight loss, and mortality risk in advanced-stage non-small cell lung cancer patients: a focus on EGFR mutation. *Nutrients* **13**, 3761 (2021). 10.3390/nu13113761 PMC8622618 34836017

[26] Hsiao, S. H. et al. Identification of subgroup patients with stage IIIB/IV non-small cell lung cancer at higher risk for brain metastases. *Lung Cancer* **82**, 319–323 (2013). 24018025 10.1016/j.lungcan.2013.08.004

[27] Li, C. et al. Osimertinib alone as second-line treatment for brain metastases (BM) control may be more limited than for non-BM in advanced NSCLC patients with an acquired EGFR T790M mutation. *Respir. Res* **22**, 145 (2021). 33975616 10.1186/s12931-021-01741-9 PMC8114713

[28] Smyth, E. N. et al. Clinicogenomic factors and treatment patterns among patients with advanced non-small cell lung cancer with or without brain metastases in the United States. *Oncologist* **28**, e1075–e1091 (2023). 37358877 10.1093/oncolo/oyad170 PMC10628559

[29] Teocharoen, R., Ruangritchankul, K., Vinayanuwattikun, C., Sriuranpong, V. & Sitthideatphaiboon, P. Vimentin expression status is a potential biomarker for brain metastasis development in EGFR-mutant NSCLC patients. *Transl. Lung Cancer Res* **10**, 790–801 (2021). 33718022 10.21037/tlcr-20-1020 PMC7947392

[30] Calle, E. E. & Kaaks, R. Overweight, obesity and cancer: epidemiological evidence and proposed mechanisms. *Nat. Rev. Cancer* **4**, 579–591 (2004). 15286738 10.1038/nrc1408

[31] Quail, D. F. et al. Obesity alters the lung myeloid cell landscape to enhance breast cancer metastasis through IL5 and GM-CSF. *Nat. Cell Biol.* **19**, 974–987 (2017). 28737771 10.1038/ncb3578 PMC6759922

[32] Abizaid, A. et al. Ghrelin modulates the activity and synaptic input organization of midbrain dopamine neurons while promoting appetite. *J. Clin. Invest* **116**, 3229–3239 (2006). 17060947 10.1172/JCI29867 PMC1618869

[33] Banks, W. A., Tschop, M., Robinson, S. M. & Heiman, M. L. Extent and direction of ghrelin transport across the blood-brain barrier is determined by its unique primary structure. *J. Pharm. Exp. Ther.* **302**, 822–827 (2002). 10.1124/jpet.102.034827 12130749

[34] Cowley, M. A. et al. The distribution and mechanism of action of ghrelin in the CNS demonstrates a novel hypothalamic circuit regulating energy homeostasis. *Neuron* **37**, 649–661 (2003). 12597862 10.1016/s0896-6273(03)00063-1

[35] Cummings, D. E. & Shannon, M. H. Roles for ghrelin in the regulation of appetite and body weight. *Arch. Surg.* **138**, 389–396 (2003). 12686525 10.1001/archsurg.138.4.389

[36] Howard, A. D. et al. A receptor in pituitary and hypothalamus that functions in growth hormone release. *Science* **273**, 974–977 (1996). 8688086 10.1126/science.273.5277.974

[37] Kojima, M. et al. Ghrelin is a growth-hormone-releasing acylated peptide from stomach. *Nature* **402**, 656–660 (1999). 10604470 10.1038/45230

[38] Nakazato, M. et al. A role for ghrelin in the central regulation of feeding. *Nature* **409**, 194–198 (2001). 11196643 10.1038/35051587

[39] Uriarte, M. et al. Evidence Supporting a Role for the Blood-Cerebrospinal Fluid Barrier Transporting Circulating Ghrelin into the Brain. *Mol. Neurobiol.* **56**, 4120–4134 (2019). 30276663 10.1007/s12035-018-1362-8

[40] Cummings, D. E. et al. A preprandial rise in plasma ghrelin levels suggests a role in meal initiation in humans. *Diabetes* **50**, 1714–1719 (2001). 11473029 10.2337/diabetes.50.8.1714

[41] De Souza, M. J., Leidy, H. J., O’Donnell, E., Lasley, B. & Williams, N. I. Fasting ghrelin levels in physically active women: relationship with menstrual disturbances and metabolic hormones. *J. Clin. Endocrinol. Metab.* **89**, 3536–3542 (2004). 15240643 10.1210/jc.2003-032007

[42] Marzullo, P. et al. The relationship between active ghrelin levels and human obesity involves alterations in resting energy expenditure. *J. Clin. Endocrinol. Metab.* **89**, 936–939 (2004). 14764817 10.1210/jc.2003-031328

[43] Shiiya, T. et al. Plasma ghrelin levels in lean and obese humans and the effect of glucose on ghrelin secretion. *J. Clin. Endocrinol. Metab.* **87**, 240–244 (2002). 11788653 10.1210/jcem.87.1.8129

[44] Ybarra, J. et al. Persistent correlation of ghrelin plasma levels with body mass index both in stable weight conditions and during gastric-bypass-induced weight loss. *Obes. Surg.* **19**, 327–331 (2009). 18975038 10.1007/s11695-008-9748-8

[45] Katsuki, A. et al. Circulating levels of active ghrelin is associated with abdominal adiposity, hyperinsulinemia and insulin resistance in patients with type 2 diabetes mellitus. *Eur. J. Endocrinol.* **151**, 573–577 (2004). 15538935 10.1530/eje.0.1510573

[46] Valera Mora, M. E. et al. Ghrelin does not influence gastric emptying in obese subjects. *Obes. Res* **13**, 739–744 (2005). 15897483 10.1038/oby.2005.83

[47] Sjostedt E. et al. An atlas of the protein-coding genes in the human, pig, and mouse brain. *Science* **367**, eaay5947 (2020). 10.1126/science.aay5947 32139519

[48] Cavalier, M. et al. Involvement of PKA and ERK pathways in ghrelin-induced long-lasting potentiation of excitatory synaptic transmission in the CA1 area of rat hippocampus. *Eur. J. Neurosci.* **42**, 2568–2576 (2015). 26153524 10.1111/ejn.13013

[49] Sieburth, D., Madison, J. M. & Kaplan, J. M. PKC-1 regulates secretion of neuropeptides. *Nat. Neurosci.* **10**, 49–57 (2007). 17128266 10.1038/nn1810

[50] Zhang, Q. et al. The spatial transcriptomic landscape of non-small cell lung cancer brain metastasis. *Nat. Commun.* **13**, 5983 (2022). 36216799 10.1038/s41467-022-33365-y PMC9551067

[51] Iwamoto, T. et al. Distinct gene expression profiles between primary breast cancers and brain metastases from pair-matched samples. *Sci. Rep.* **9**, 13343 (2019). 31527824 10.1038/s41598-019-50099-y PMC6746866

[52] Marzese, D. M. et al. Epigenome-wide DNA methylation landscape of melanoma progression to brain metastasis reveals aberrations on homeobox D cluster associated with prognosis. *Hum. Mol. Genet* **23**, 226–238 (2014). 24014427 10.1093/hmg/ddt420 PMC3857956

[53] Chen, G. et al. Molecular profiling of patient-matched brain and extracranial melanoma metastases implicates the PI3K pathway as a therapeutic target. *Clin. Cancer Res* **20**, 5537–5546 (2014). 24803579 10.1158/1078-0432.CCR-13-3003 PMC4216765

[54] Hollingshead, M. G. et al. Gene expression profiling of 49 human tumor xenografts from in vitro culture through multiple in vivo passages–strategies for data mining in support of therapeutic studies. *BMC Genomics* **15**, 393 (2014). 24885658 10.1186/1471-2164-15-393 PMC4041995

[55] Laughney, A. M. et al. Regenerative lineages and immune-mediated pruning in lung cancer metastasis. *Nat. Med* **26**, 259–269 (2020). 32042191 10.1038/s41591-019-0750-6 PMC7021003

[56] Zhang, X. H. et al. Latent bone metastasis in breast cancer tied to Src-dependent survival signals. *Cancer Cell* **16**, 67–78 (2009). 19573813 10.1016/j.ccr.2009.05.017 PMC2749247

[57] Dietrich, P. et al. Molecular crosstalk between Y5 receptor and neuropeptide Y drives liver cancer. *J. Clin. Invest* **130**, 2509–2526 (2020). 31999643 10.1172/JCI131919 PMC7190991

[58] Lu, C. et al. Hypoxia-activated neuropeptide Y/Y5 receptor/RhoA pathway triggers chromosomal instability and bone metastasis in Ewing sarcoma. *Nat. Commun.* **13**, 2323 (2022). 35484119 10.1038/s41467-022-29898-x PMC9051212

[59] Dumont, Y., Fournier, A. & Quirion, R. Expression and characterization of the neuropeptide Y Y5 receptor subtype in the rat brain. *J. Neurosci.* **18**, 5565–5574 (1998). 9671648 10.1523/JNEUROSCI.18-15-05565.1998 PMC6793038

[60] Nichol, K. A. et al. Conservation of expression of neuropeptide Y5 receptor between human and rat hypothalamus and limbic regions suggests an integral role in central neuroendocrine control. *J. Neurosci.* **19**, 10295–10304 (1999). 10575027 10.1523/JNEUROSCI.19-23-10295.1999 PMC6782429

[61] Subramanian, A. et al. Gene set enrichment analysis: a knowledge-based approach for interpreting genome-wide expression profiles. *Proc. Natl Acad. Sci. USA* **102**, 15545–15550 (2005). 16199517 10.1073/pnas.0506580102 PMC1239896

[62] Wu, Y., Berisha, A. & Borniger, J. C. Neuropeptides in Cancer: Friend and Foe?. *Adv. Biol. (Weinh.)* **6**, e2200111 (2022). 35775608 10.1002/adbi.202200111

[63] Yi, M. et al. A Promising Therapeutic Target for Metabolic Diseases: Neuropeptide Y Receptors in Humans. *Cell Physiol. Biochem* **45**, 88–107 (2018). 29310113 10.1159/000486225

[64] Lehuede, C., Dupuy, F., Rabinovitch, R., Jones, R. G. & Siegel, P. M. Metabolic Plasticity as a Determinant of Tumor Growth and Metastasis. *Cancer Res* **76**, 5201–5208 (2016). 27587539 10.1158/0008-5472.CAN-16-0266

[65] Tyagi, A., Wu, S. Y. & Watabe, K. Metabolism in the progression and metastasis of brain tumors. *Cancer Lett.* **539**, 215713 (2022). 35513201 10.1016/j.canlet.2022.215713 PMC9999298

[66] Shimano, H. & Sato, R. SREBP-regulated lipid metabolism: convergent physiology - divergent pathophysiology. *Nat. Rev. Endocrinol.* **13**, 710–730 (2017). 28849786 10.1038/nrendo.2017.91

[67] Woldmar, N. et al. Proteomic analysis of brain metastatic lung adenocarcinoma reveals intertumoral heterogeneity and specific alterations associated with the timing of brain metastases. *ESMO Open* **8**, 100741 (2023). 36527824 10.1016/j.esmoop.2022.100741 PMC10024110

[68] Cheng, C. et al. Ammonia stimulates SCAP/Insig dissociation and SREBP-1 activation to promote lipogenesis and tumour growth. *Nat. Metab.* **4**, 575–588 (2022). 35534729 10.1038/s42255-022-00568-y PMC9177652

[69] Erondu, N. et al. Neuropeptide Y5 receptor antagonism does not induce clinically meaningful weight loss in overweight and obese adults. *Cell Metab.* **4**, 275–282 (2006). 17011500 10.1016/j.cmet.2006.08.002

[70] Erondu, N. et al. Effect of NPY5R antagonist MK-0557 on weight regain after very-low-calorie diet-induced weight loss. *Obes. (Silver Spring)* **15**, 895–905 (2007). 10.1038/oby.2007.620 17426325

[71] Arnold, M. et al. Global burden of cancer attributable to high body-mass index in 2012: a population-based study. *Lancet Oncol.* **16**, 36–46 (2015). 25467404 10.1016/S1470-2045(14)71123-4 PMC4314462

[72] Bhaskaran, K. et al. Body-mass index and risk of 22 specific cancers: a population-based cohort study of 5.24 million UK adults. *Lancet* **384**, 755–765 (2014). 25129328 10.1016/S0140-6736(14)60892-8 PMC4151483

[73] Flegal, K. M., Kit, B. K., Orpana, H. & Graubard, B. I. Association of all-cause mortality with overweight and obesity using standard body mass index categories: a systematic review and meta-analysis. *JAMA* **309**, 71–82 (2013). 23280227 10.1001/jama.2012.113905 PMC4855514

[74] Recalde, M. et al. Body mass index and waist circumference in relation to the risk of 26 types of cancer: a prospective cohort study of 3.5 million adults in Spain. *BMC Med* **19**, 10 (2021). 33441148 10.1186/s12916-020-01877-3 PMC7807518

[75] Gupta, A. et al. Premorbid body mass index and mortality in patients with lung cancer: A systematic review and meta-analysis. *Lung Cancer* **102**, 49–59 (2016). 27987589 10.1016/j.lungcan.2016.10.017

[76] Jiang, M. et al. The relationship between body-mass index and overall survival in non-small cell lung cancer by sex, smoking status, and race: A pooled analysis of 20,937 International lung Cancer consortium (ILCCO) patients. *Lung Cancer* **152**, 58–65 (2021). 33352384 10.1016/j.lungcan.2020.11.029 PMC8042597

[77] Smith, L. et al. Body mass index and risk of lung cancer among never, former, and current smokers. *J. Natl Cancer Inst.* **104**, 778–789 (2012). 22457475 10.1093/jnci/djs179 PMC3352831

[78] Dahlberg, S. E. et al. Body mass index and its association with clinical outcomes for advanced non-small-cell lung cancer patients enrolled on Eastern Cooperative Oncology Group clinical trials. *J. Thorac. Oncol.* **8**, 1121–1127 (2013). 23887169 10.1097/JTO.0b013e31829cf942 PMC3763835

[79] Lam, V. K. et al. Obesity is associated with long-term improved survival in definitively treated locally advanced non-small cell lung cancer (NSCLC). *Lung Cancer* **104**, 52–57 (2017). 28213000 10.1016/j.lungcan.2016.11.017

[80] Lennon, H., Sperrin, M., Badrick, E. & Renehan, A. G. The Obesity Paradox in Cancer: a Review. *Curr. Oncol. Rep.* **18**, 56 (2016). 27475805 10.1007/s11912-016-0539-4 PMC4967417

[81] Nitsche L. J. et al. Exploring the Impact of the Obesity Paradox on Lung Cancer and Other Malignancies. *Cancers (Basel)* **14**, (2022). 10.3390/cancers14061440 PMC8946288 35326592

[82] Han, J. et al. Imaging modalities for diagnosis and monitoring of cancer cachexia. *EJNMMI Res* **11**, 94 (2021). 34557972 10.1186/s13550-021-00834-2 PMC8460705

[83] Hall, K. D. et al. The energy balance model of obesity: beyond calories in, calories out. *Am. J. Clin. Nutr.* **115**, 1243–1254 (2022). 35134825 10.1093/ajcn/nqac031 PMC9071483

[84] Ulmann, G. et al. Lean Body Mass and Endocrine Status But Not Age Are Determinants of Resting Energy Expenditure in Patients with Non-Small Cell Lung Cancer. *Ann. Nutr. Metab.* **75**, 223–230 (2019). 31865308 10.1159/000504874

[85] Hu, S. et al. Higher than predicted resting energy expenditure and lower physical activity in healthy underweight Chinese adults. *Cell Metab.* **34**, 1413–1415 (2022). 35839758 10.1016/j.cmet.2022.05.012

[86] Jouinot, A. et al. Hypermetabolism is an independent prognostic factor of survival in metastatic non-small cell lung cancer patients. *Clin. Nutr.* **39**, 1893–1899 (2020). 31443979 10.1016/j.clnu.2019.08.003

[87] Wren, A. M. et al. The novel hypothalamic peptide ghrelin stimulates food intake and growth hormone secretion. *Endocrinology* **141**, 4325–4328 (2000). 11089570 10.1210/endo.141.11.7873

[88] Schaeffer, M. et al. Rapid sensing of circulating ghrelin by hypothalamic appetite-modifying neurons. *Proc. Natl Acad. Sci. USA* **110**, 1512–1517 (2013). 23297228 10.1073/pnas.1212137110 PMC3557016

[89] Kim, Y. et al. Ghrelin is required for dietary restriction-induced enhancement of hippocampal neurogenesis: lessons from ghrelin knockout mice. *Endocr. J.* **62**, 269–275 (2015). 25735661 10.1507/endocrj.EJ14-0436

[90] Sassi, M., Morgan, A. H. & Davies, J. S. Ghrelin acylation-a post-translational tuning mechanism regulating adult hippocampal neurogenesis. *Cells* **11**, 765 (2022). 10.3390/cells11050765 PMC8909677 35269387

[91] Frago, L. M., Chowen, J. A. Involvement of astrocytes in mediating the central effects of ghrelin. *Int. J. Mol. Sci.* **18**, 536 (2017). 10.3390/ijms18030536 PMC5372552 28257088

[92] Lee, J. Y. & Yune, T. Y. Ghrelin inhibits oligodendrocyte cell death by attenuating microglial activation. *Endocrinol. Metab. (Seoul.)* **29**, 371–378 (2014). 25309797 10.3803/EnM.2014.29.3.371 PMC4192814

[93] Moon, M. et al. Neuroprotective effect of ghrelin in the 1-methyl-4-phenyl-1,2,3,6-tetrahydropyridine mouse model of Parkinson’s disease by blocking microglial activation. *Neurotox. Res* **15**, 332–347 (2009). 19384567 10.1007/s12640-009-9037-x

[94] Tian R. & Mao G. Ghrelin reduces cerebral ischemic injury in rats by reducing M1 microglia/macrophages. *Eur. J. Histochem.* **66**, 3350 (2022). 10.4081/ejh.2022.3350 PMC8764466 35016495

[95] Diano, S. et al. Ghrelin controls hippocampal spine synapse density and memory performance. *Nat. Neurosci.* **9**, 381–388 (2006). 16491079 10.1038/nn1656

[96] Chuang, J. C., Zigman, J. M. Ghrelin’s roles in stress, mood, and anxiety regulation. *Int. J. Pept.* **2010**, 460549 (2010). 10.1155/2010/460549 PMC2915752 20721341

[97] Lutter, M. et al. The orexigenic hormone ghrelin defends against depressive symptoms of chronic stress. *Nat. Neurosci.* **11**, 752–753 (2008). 18552842 10.1038/nn.2139 PMC2765052

[98] Dixit, V. D. et al. Ghrelin promotes thymopoiesis during aging. *J. Clin. Invest* **117**, 2778–2790 (2007). 17823656 10.1172/JCI30248 PMC1964507

[99] Blauwhoff-Buskermolen, S. et al. Plasma ghrelin levels are associated with anorexia but not cachexia in patients with NSCLC. *Front Physiol.* **8**, 119 (2017). 28298897 10.3389/fphys.2017.00119 PMC5331052

[100] Karapanagiotou, E. M. et al. Increased serum levels of ghrelin at diagnosis mediate body weight loss in non-small cell lung cancer (NSCLC) patients. *Lung Cancer* **66**, 393–398 (2009). 19282046 10.1016/j.lungcan.2009.02.006

[101] Leidy, H. J., Dougherty, K. A., Frye, B. R., Duke, K. M. & Williams, N. I. Twenty-four-hour ghrelin is elevated after calorie restriction and exercise training in non-obese women. *Obes. (Silver Spring)* **15**, 446–455 (2007). 10.1038/oby.2007.542 17299118

[102] Papandreou, D., Karavolias, C., Arvaniti, F., Kafeza, E. & Sidawi, F. Fasting ghrelin levels are decreased in obese subjects and are significantly related with insulin resistance and body mass index. *Open Access Maced. J. Med Sci.* **5**, 699–702 (2017). 29104675 10.3889/oamjms.2017.182 PMC5661704

[103] Tschop, M. et al. Circulating ghrelin levels are decreased in human obesity. *Diabetes* **50**, 707–709 (2001). 11289032 10.2337/diabetes.50.4.707

[104] Au C. C. et al. Three-dimensional growth of breast cancer cells potentiates the anti-tumor effects of unacylated ghrelin and AZP-531. *Elife* **9**, e56913(2020). 10.7554/eLife.56913 PMC7363447 32667883

[105] Kawaguchi, M. et al. Ghrelin administration suppresses inflammation-associated colorectal carcinogenesis in mice. *Cancer Sci.* **106**, 1130–1136 (2015). 26094822 10.1111/cas.12725 PMC4582981

[106] Lawnicka, H., Melen-Mucha, G., Motylewska, E., Mucha, S. & Stepien, H. Modulation of ghrelin axis influences the growth of colonic and prostatic cancer cells in vitro. *Pharm. Rep.* **64**, 951–959 (2012). 10.1016/s1734-1140(12)70890-3 23087147

[107] Cornejo, M. P. et al. Ghrelin receptor signaling targets segregated clusters of neurons within the nucleus of the solitary tract. *Brain Struct. Funct.* **223**, 3133–3147 (2018). 29761230 10.1007/s00429-018-1682-5

[108] Goldstein, J. L. et al. Surviving starvation: essential role of the ghrelin-growth hormone axis. *Cold Spring Harb. Symp. Quant. Biol.* **76**, 121–127 (2011). 21785007 10.1101/sqb.2011.76.010447

[109] Hornsby, A. K. et al. Short-term calorie restriction enhances adult hippocampal neurogenesis and remote fear memory in a Ghsr-dependent manner. *Psychoneuroendocrinology* **63**, 198–207 (2016). 26460782 10.1016/j.psyneuen.2015.09.023 PMC4686051

[110] Wu, C. S. et al. Ghrelin receptor in agouti-related peptide neurones regulates metabolic adaptation to calorie restriction. *J. Neuroendocrinol.* **31**, e12763 (2019). 31251830 10.1111/jne.12763 PMC7233797

[111] Chen, S. et al. Ghrelin is a possible new predictor associated with executive function in patients with type 2 diabetes mellitus. *J. Diabetes Investig.* **8**, 306–313 (2017). 27689345 10.1111/jdi.12580 PMC5415456

[112] Doogue, M. P. et al. Metformin increases plasma ghrelin in Type 2 diabetes. *Br. J. Clin. Pharm.* **68**, 875–882 (2009). 10.1111/j.1365-2125.2009.03372.x PMC2810798 20002081

[113] English, P. J. et al. Metformin prolongs the postprandial fall in plasma ghrelin concentrations in type 2 diabetes. *Diabetes Metab. Res Rev.* **23**, 299–303 (2007). 16952199 10.1002/dmrr.681

[114] Ida, S., Murata, K. & Kaneko, R. Effects of metformin treatment on blood leptin and ghrelin levels in patients with type 2 diabetes mellitus. *J. Diabetes* **9**, 526–535 (2017). 27380451 10.1111/1753-0407.12445

[115] Poykko, S. M. et al. Low plasma ghrelin is associated with insulin resistance, hypertension, and the prevalence of type 2 diabetes. *Diabetes* **52**, 2546–2553 (2003). 14514639 10.2337/diabetes.52.10.2546

[116] Augustin, R. C. et al. Metformin is associated with improved clinical outcomes in patients with melanoma: a retrospective, multi-institutional study. *Front Oncol.* **13**, 1075823 (2023). 37397389 10.3389/fonc.2023.1075823 PMC10312386

[117] LeCompte, M. C. et al. Impact of Diabetes Mellitus and Metformin on Clinical Outcomes of Brain Metastasis Patients Treated with Stereotactic Radiosurgery. *Int. J. Radiat. Oncol., Biol., Phys.* **102**, e328 (2018).

[118] Kang, J. et al. The associations of aspirin, statins, and metformin with lung cancer risk and related mortality: a time-dependent analysis of population-based nationally representative data. *J. Thorac. Oncol.* **16**, 76–88 (2021). 32950701 10.1016/j.jtho.2020.08.021

[119] Tseng, C. H. Metformin and lung cancer risk in patients with type 2 diabetes mellitus. *Oncotarget* **8**, 41132–41142 (2017). 28456789 10.18632/oncotarget.17066 PMC5522244

[120] Xiao, K. et al. The effect of metformin on lung cancer risk and survival in patients with type 2 diabetes mellitus: A meta-analysis. *J. Clin. Pharm. Ther.* **45**, 783–792 (2020). 32406122 10.1111/jcpt.13167

[121] Yao, L. et al. Metformin use and lung cancer risk in diabetic patients: a systematic review and meta-analysis. *Dis. Markers* **2019**, 6230162 (2019). 30881522 10.1155/2019/6230162 PMC6387718

[122] Hahn, T. M., Breininger, J. F., Baskin, D. G. & Schwartz, M. W. Coexpression of Agrp and NPY in fasting-activated hypothalamic neurons. *Nat. Neurosci.* **1**, 271–272 (1998). 10195157 10.1038/1082

[123] Nergardh, R. et al. Neuropeptide Y facilitates activity-based-anorexia. *Psychoneuroendocrinology* **32**, 493–502 (2007). 17467917 10.1016/j.psyneuen.2007.03.002

[124] Ruegsegger, G. N. et al. Hypothalamic Npy mRNA is correlated with increased wheel running and decreased body fat in calorie-restricted rats. *Neurosci. Lett.* **618**, 83–88 (2016). 26921453 10.1016/j.neulet.2016.02.037

[125] Yoshihara, T., Honma, S. & Honma, K. Effects of restricted daily feeding on neuropeptide Y release in the rat paraventricular nucleus. *Am. J. Physiol.* **270**, E589–E595 (1996). 8928763 10.1152/ajpendo.1996.270.4.E589

[126] Ferreira-Marques, M., Carvalho, A., Cavadas, C. & Aveleira, C. A. PI3K/AKT/MTOR and ERK1/2-MAPK signaling pathways are involved in autophagy stimulation induced by caloric restriction or caloric restriction mimetics in cortical neurons. *Aging (Albany NY)* **13**, 7872–7882 (2021). 33714946 10.18632/aging.202805 PMC8034898

[127] Dube, M. G., Xu, B., Crowley, W. R., Kalra, P. S. & Kalra, S. P. Evidence that neuropeptide Y is a physiological signal for normal food intake. *Brain Res* **646**, 341–344 (1994). 8069687 10.1016/0006-8993(94)90103-1

[128] Loh, K., Herzog, H. & Shi, Y. C. Regulation of energy homeostasis by the NPY system. *Trends Endocrinol. Metab.* **26**, 125–135 (2015). 25662369 10.1016/j.tem.2015.01.003

[129] Czarnecka, M. et al. Neuropeptide Y receptor Y5 as an inducible pro-survival factor in neuroblastoma: implications for tumor chemoresistance. *Oncogene* **34**, 3131–3143 (2015). 25132261 10.1038/onc.2014.253 PMC4333135

[130] Dvorakova, M. C., Kruzliak, P. & Rabkin, S. W. Role of neuropeptides in cardiomyopathies. *Peptides* **61**, 1–6 (2014). 25149360 10.1016/j.peptides.2014.08.004

[131] Qin, Y. Y. et al. Neuropeptide Y attenuates cardiac remodeling and deterioration of function following myocardial infarction. *Mol. Ther.* **32**, 559–561 (2024). 38118444 10.1016/j.ymthe.2023.12.013 PMC10861985

[132] Ciminera, A. K., Jandial, R. & Termini, J. Metabolic advantages and vulnerabilities in brain metastases. *Clin. Exp. Metastasis* **34**, 401–410 (2017). 29063238 10.1007/s10585-017-9864-8 PMC5712254

[133] Schild, T., Low, V., Blenis, J. & Gomes, A. P. Unique metabolic adaptations dictate distal organ-specific metastatic colonization. *Cancer Cell* **33**, 347–354 (2018). 29533780 10.1016/j.ccell.2018.02.001 PMC5889305

[134] Huang, Y., Lin, X. & Lin, S. Neuropeptide Y and metabolism syndrome: an update on perspectives of clinical therapeutic intervention strategies. *Front Cell Dev. Biol.* **9**, 695623 (2021). 34307371 10.3389/fcell.2021.695623 PMC8299562

[135] Cheng, Y. J., Fan, F., Zhang, Z. & Zhang, H. J. Lipid metabolism in malignant tumor brain metastasis: reprogramming and therapeutic potential. *Expert Opin. Ther. Targets* **27**, 861–878 (2023). 37668244 10.1080/14728222.2023.2255377

[136] Currie, E., Schulze, A., Zechner, R., Walther, T. C. & Farese, R. V. Jr. Cellular fatty acid metabolism and cancer. *Cell Metab.* **18**, 153–161 (2013). 23791484 10.1016/j.cmet.2013.05.017 PMC3742569

[137] Duman, C. et al. Acyl-CoA-binding protein drives glioblastoma tumorigenesis by sustaining fatty acid oxidation. *Cell Metab.* **30**, 274–289 e275 (2019). 31056285 10.1016/j.cmet.2019.04.004

[138] Ferraro, G. B. et al. Fatty acid synthesis is required for breast cancer brain metastasis. *Nat. Cancer* **2**, 414–428 (2021). 34179825 10.1038/s43018-021-00183-y PMC8223728

[139] McKelvey, K. J. et al. Glycolysis and Fatty Acid Oxidation Inhibition Improves Survival in Glioblastoma. *Front Oncol.* **11**, 633210 (2021). 33854970 10.3389/fonc.2021.633210 PMC8039392

[140] Sperry, J. et al. Glioblastoma utilizes fatty acids and ketone bodies for growth allowing progression during ketogenic diet therapy. *iScience* **23**, 101453 (2020). 32861192 10.1016/j.isci.2020.101453 PMC7471621

[141] Zou, Y. et al. Polyunsaturated fatty acids from astrocytes activate PPARgamma signaling in cancer cells to promote brain metastasis. *Cancer Discov.* **9**, 1720–1735 (2019). 31578185 10.1158/2159-8290.CD-19-0270 PMC6891206

[142] Deng, Q. et al. Proteomics-based model for predicting the risk of brain metastasis in patients with resected lung adenocarcinoma carrying the EGFR mutation. *Int J. Med Sci.* **21**, 765–774 (2024). 38464823 10.7150/ijms.92993 PMC10920840

[143] Bruss, M. D., Khambatta, C. F., Ruby, M. A., Aggarwal, I. & Hellerstein, M. K. Calorie restriction increases fatty acid synthesis and whole body fat oxidation rates. *Am. J. Physiol. Endocrinol. Metab.* **298**, E108–E116 (2010). 19887594 10.1152/ajpendo.00524.2009 PMC4056782

[144] Mihaylova, M. M. et al. Fasting activates fatty acid oxidation to enhance intestinal stem cell function during homeostasis and aging. *Cell Stem Cell* **22**, 769–778.e764 (2018). 29727683 10.1016/j.stem.2018.04.001 PMC5940005

[145] Chen, F., Zhou, Y., Yang, K., Shen, M. & Wang, Y. NPY stimulates cholesterol synthesis acutely by activating the SREBP2-HMGCR pathway through the Y1 and Y5 receptors in murine hepatocytes. *Life Sci.* **262**, 118478 (2020). 32976883 10.1016/j.lfs.2020.118478

[146] Fukasaka, Y. et al. An insurmountable NPY Y5 receptor antagonist exhibits superior anti-obesity effects in high-fat diet-induced obese mice. *Neuropeptides* **70**, 55–63 (2018). 29801968 10.1016/j.npep.2018.05.006

[147] Ishihara, A. et al. A neuropeptide Y Y5 antagonist selectively ameliorates body weight gain and associated parameters in diet-induced obese mice. *Proc. Natl Acad. Sci. USA* **103**, 7154–7158 (2006). 16636293 10.1073/pnas.0510320103 PMC1459032

[148] Phelps R. M. et al. NCI-navy medical oncology branch cell line data base. *J. Cell Biochem*. **24**, 32–91 (1996). 10.1002/jcb.240630505 8806092

[149] Wu S. Y. et al. Nicotine promotes brain metastasis by polarizing microglia and suppressing innate immune function. *J. Exp. Med.* **217**, e20191131 (2020). 10.1084/jem.20191131 PMC7398164 32496556

[150] Xing, F. et al. Loss of XIST in breast cancer activates MSN-c-met and reprograms microglia via exosomal miRNA to promote brain metastasis. *Cancer Res* **78**, 4316–4330 (2018). 30026327 10.1158/0008-5472.CAN-18-1102 PMC6072593

[151] Wingrove, E. et al. Transcriptomic hallmarks of tumor plasticity and stromal interactions in brain metastasis. *Cell Rep.* **27**, 1277–1292.e1277 (2019). 31018140 10.1016/j.celrep.2019.03.085 PMC6592283

[152] Gyorffy, B. et al. An online survival analysis tool to rapidly assess the effect of 22,277 genes on breast cancer prognosis using microarray data of 1809 patients. *Breast Cancer Res Treat.* **123**, 725–731 (2010). 20020197 10.1007/s10549-009-0674-9

[153] Gene Ontology C. et al. The gene ontology knowledgebase in 2023. *Genetics* **224**, iyad031 (2023).

