# Supplementary material for: Ghrelin-induced neuronal NPY promotes brain metastasis in lung cancer patients with low BMI
Source: Nat Commun. 2025 Jul 1;16:5608. doi: 10.1038/s41467-025-60730-4 (PMC12218320; doi:10.1038/s41467-025-60730-4)
Supplement: Supplementary file 1 — Supplementary Information [file 41467_2025_60730_MOESM1_ESM.pdf]

Supplementary Information

For

Ghrelin-induced neuronal NPY promotes brain metastasis in lung cancer patients with low BMI

Abhishek Tyagi[1], Shih-Ying Wu[1], Jee-Won Kim[1], Ravindra Pramod Deshpande[1], Kerui Wu[2], Eleanor C. Smith[1], Giuseppe L. Banna[3] and Kounosuke Watabe[1]*

[1]Department of Cancer Biology, Wake Forest University School of Medicine, Winston-Salem, NC 27157, USA
[2]Nanoscience Department, Joint School of Nanoscience and Nanoengineering, Greensboro, NC 27401, USA
[3]Portsmouth Hospitals University NHS Trust, Faculty of Science and Health, School of Pharmacy and Biomedical Sciences, University of Portsmouth, UK

*Corresponding author(s):

Kounosuke Watabe Ph.D.
Department of Cancer Biology, Wake Forest University School of Medicine, Winston Salem, NC 27157, USA

Email: kwatabe@wakehealth.edu

Supplementary Figures 1-8
Supplementary Tables 1-3

Supplementary Figure 1

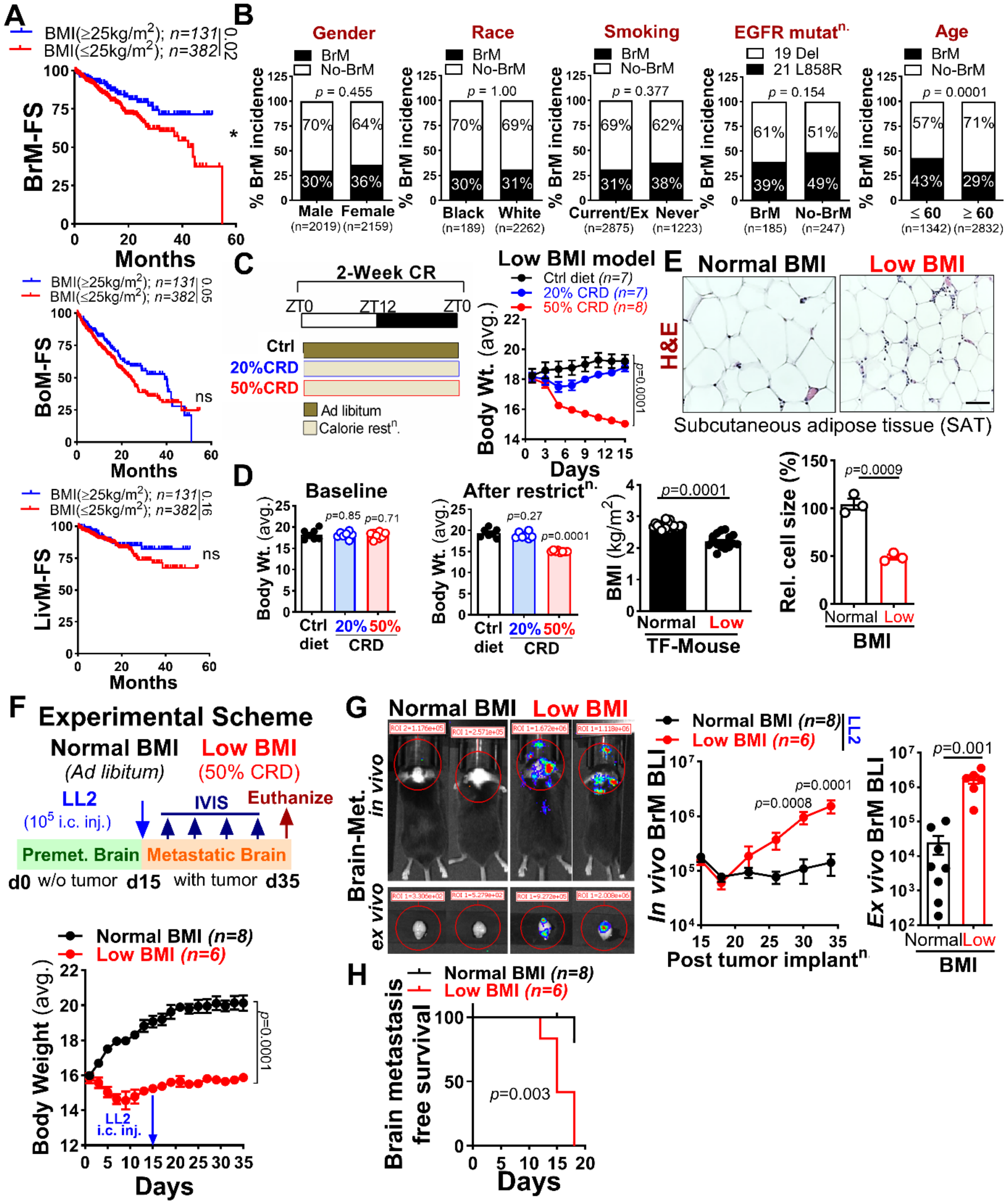

Supplementary Figure 1: Brain metastasis in lung cancer is promoted by low BMI. **A.** Kaplan–Meier analysis of organ-specific metastasis-free survival in metastatic lung cancer patients with BMI status (*n=513*; log-rank [Mantel–Cox] test). **B.** Incidence of brain metastasis in a metastatic lung cancer patient cohort, stratified by gender, race, smoking, EGFR mutation and age (two-sided Fisher-exact test). **C.** Left panel: Schematic representation of the study design. Right panel: Average body weight measurement in each group during the study (two-way ANOVA with Tukey's test). **D.** Left and middle panel: Comparison of average body weight of groups before (baseline) and after two-weeks of calorie restriction with time-restricted feeding (CRD; unpaired two-tailed t-test). Right panel: BMI quantification of mice across the study duration (unpaired two-tailed t-test). **E.** Upper panel: Representative microphotographs of H&E-stained sections of subcutaneous adipose tissue (SAT) from normal and low BMI mice after 2-weeks of CRD (Scale bar: 100 μm). Lower panel: Quantitative analysis of adipocyte cell size in the SAT samples (*n=4* independent experiments; unpaired two-tailed t-test). **F.** Upper panel: Schematic diagram of the metastasis assay. C57BL/6 mice were fed either a normal (*ad-libitum*) or 50% calorie-restricted diet (12-hr/dark phase) until day 15 to establish low BMI mice, followed by intracardiac injection (i.c.) of LL/2 murine lung cancer cells (10^5). Lower panel: Average body weight curve for each group over the study duration (two-way ANOVA with Tukey's test). **G.** Left panel: Representative *in vivo* (top) and *ex vivo* images of brain metastasis (bottom) at the endpoint. Middle and right panel: *In vivo* and *ex vivo* quantification of brain metastasis in normal (*n=8*) and low BMI (*n=6*) mice by BLI at the endpoint (two-way ANOVA with Tukey's test (*in vivo*); unpaired two-tailed t-test (*ex vivo*). **H.** Kaplan–Meier analysis of brain metastasis-free survival in normal (*n=8*) and low BMI (*n=6*) mice [log-rank (Mantel–Cox test)]. All data are mean ± S.E.M. Source data are provided as a Source Data file.

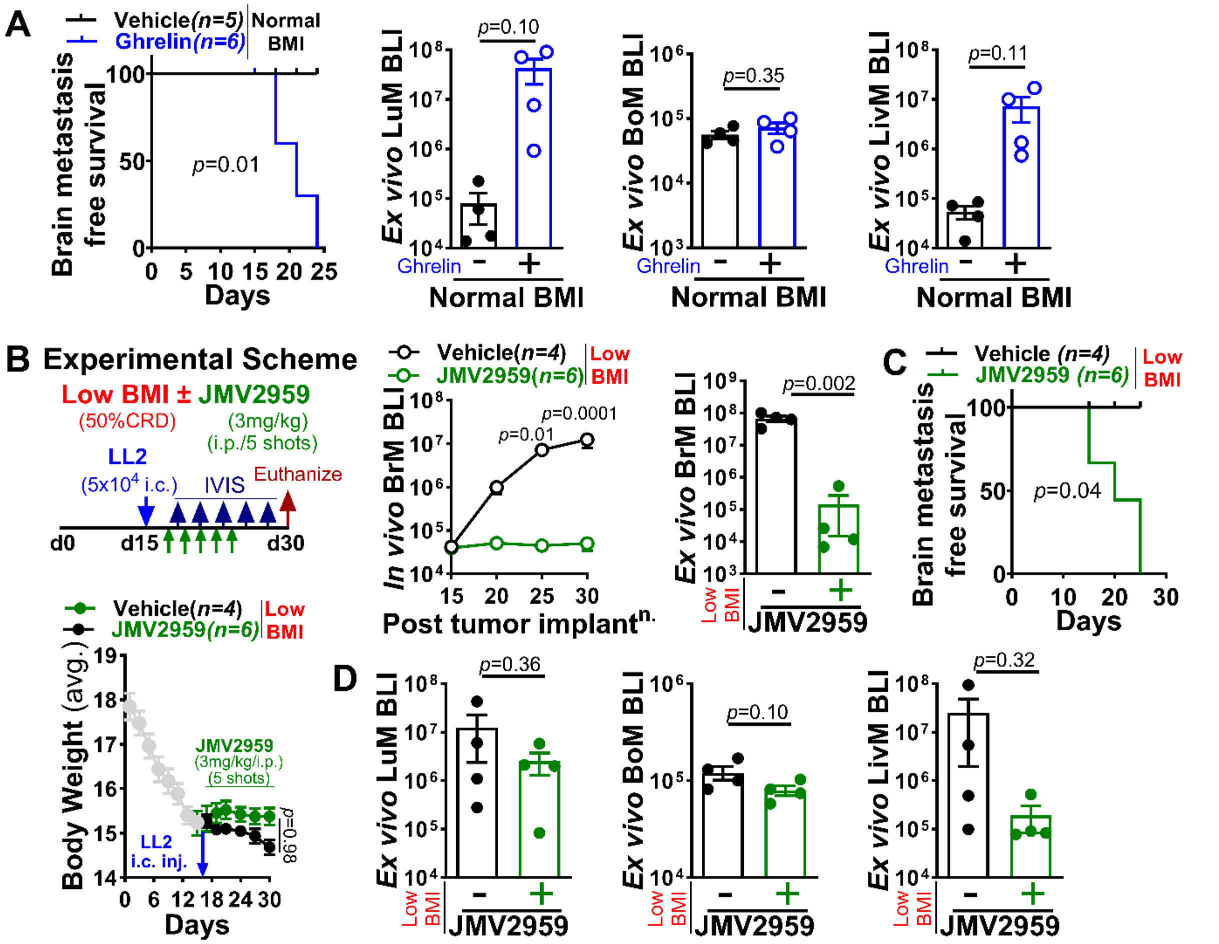

Supplementary Figure 2: Ghrelin receptor inhibition abrogates low BMI induced brain metastasis. A. Left panel: Brain metastasis-free survival of control (*n=5*) and ghrelin-treated normal BMI mice (*n=6*) as analyzed by Kaplan–Meier plot (log-rank [Mantel–Cox] test). Right panel: *Ex vivo* quantification of metastasis in lung, liver and bone in control and ghrelin-treated normal BMI mice by BLI at the endpoint (*n=4/*group each, unpaired two-tailed t-test). **B.** Upper left panel: Schematic of the metastasis assay. Immune-competent C57BL/6 mice were fed with a 50% calorie restricted diet (12-hr/dark phase) for 15 days, followed by administration of the ghrelin receptor inhibitor (JMV2959; 3mg/kg; 5 shots; i.p.) post-intracardiac injection of LL2 lung cancer cells (5x10^4). Lower left panel: Average body weight curve of each group throughout the study (two-way ANOVA with Tukey's test). Upper right panel: *In vivo* and *ex vivo* quantification of brain metastasis in control (*n=4*) and ghrelin receptor inhibitor-treated (*n=6*) low BMI mice by

BLI at the endpoint (two-way ANOVA with Tukey's test (*in vivo*); unpaired two-tailed t-test (*ex vivo*). **C.** Kaplan–Meier analysis of brain metastasis-free survival in control (*n=4*) and ghrelin receptor inhibitor-treated low BMI mice (*n=6*) [log-rank (Mantel–Cox test)]. **D.** *Ex vivo* quantification of metastasis in lung, liver and bone in control and ghrelin receptor inhibitor-treated low BMI mice (*n=4/*group) by BLI at the endpoint (unpaired two-tailed t-test). All data are mean ± S.E.M. Source data are provided as a Source Data file.

Supplementary Figure 3

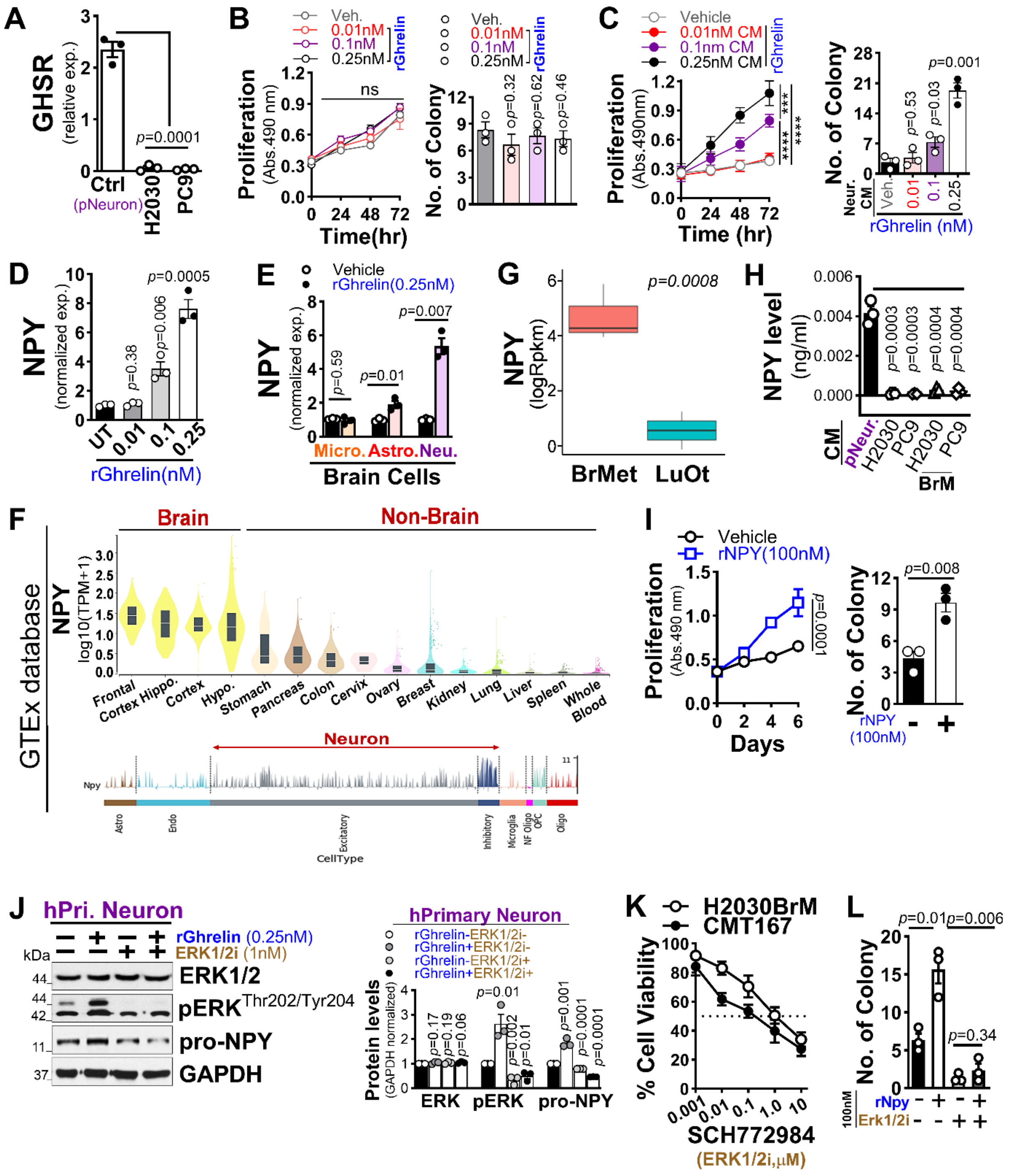

Supplementary Figure 3: Low BMI-induced ghrelin activates neuronal NPY and promotes tumor growth. A. Relative expression of ghrelin receptor (GHSR) in parental lung cancer cell lines (H2030, PC9) compared to human primary neurons (control) by qRT-PCR, normalized to β-Actin (*n=3* independent experiments, unpaired two-tailed t-test). **B.** H2030BrM cells were treated with vehicle or rGhrelin protein (0.01 nM-0.25 nM) and examined for cell proliferation by MTS assay (left) and growth (right) by colony formation assay (*n=3* independent experiments, unpaired two-tailed t-test). **C.** H2030BrM cancer cells were treated with or without CM from ghrelin-pretreated neurons (0.01nM-0.25nM) and examined for cell proliferation by MTS assay (left) and growth (right) by colony formation assay (*n=3* independent experiments, unpaired two-tailed t-test). **D.** NPY expression in human primary neurons treated with or without rGhrelin (0.01 nM-0.25 nM; 12 hr) by qRT-PCR, normalized to β-Actin (*n=3* independent experiments, unpaired two-tailed t-test). **E.** NPY expression in human brain cells treated with or without dose-titrated rGhrelin (0.25nM) by qRT-PCR, normalized to β-Actin (*n=3* independent experiments, unpaired two-tailed t-test). **F.** NPY expression as log transcript per million (TPM+1) across various tissues, including brain and non-brain, and brain cell types, using the GTEx database. **G.** Differential expression of NPY in brain metastases versus primary lung tumors from H2030BrM xenografts, using GEO dataset (GSE115699). **H.** Quantification of NPY protein levels in CM from parental and brain-tropic lung cancer cell lines by ELISA (*n= 3* independent experiments, unpaired two-tailed t-test). **I.** PC9BrM cells were treated with or without rNPY (100nM) and examined for cell proliferation by MTS assay (left) and growth (right) by colony formation assay (*n=3* independent experiments, lower panel; unpaired two-tailed t-test). **J.** Western blot (right) and ImageJ quantification (left) of GHSR signaling proteins in primary neurons treated with or without rGhrelin (0.25 nM) in the presence or absence of ERK inhibitor (ERK1/2i, SCH772984,1nM). Normalization to GAPDH (*n=3* independent experiments; unpaired two-tailed t-test). **K.** Dose-dependent effect of ERK1/2i on H2030BrM and CMT167 cancer cells viability as examined by MTT assay (*n=3* independent experiments). **L.** CMT167 cancer cells were treated with or without rNPY protein (100 nM, 12 hr) in presence or absence of Erk1/2i (100 nM) and examined for growth by colony formation assay (*n=3* independent experiments, unpaired two-tailed t-test). All data are mean ± S.E.M. Source data are

provided as a Source Data file.

Supplementary Figure 4

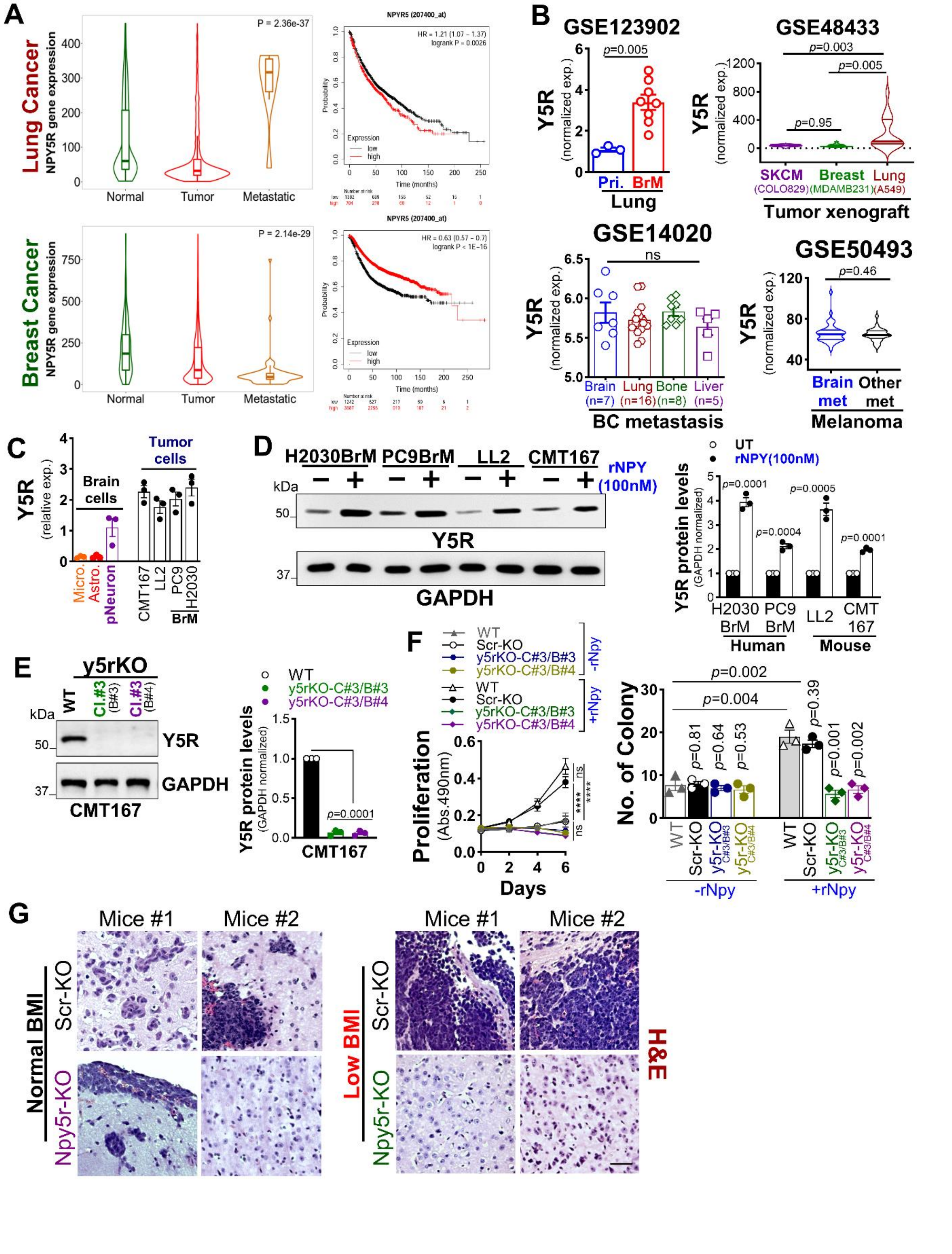

Supplementary Figure 4: Neuronal NPY enhances brain metastatic growth via Y5R receptor signaling. A. Left panels: Representative violin plots showing differential expression of Y5R receptor expression in normal tissues (*n=391; n=242*), primary tumors (*n=1865; n=7,569*) and metastatic tumors (*n=8; n=82*) from lung and breast cancer patients. Right panels: Kaplan–Meier survival analysis comparing overall survival probabilities of lung (*n=2,166*) and breast (*n=1,879*) primary cancer patients with high and low Y5R receptor expression. **B.** Violin and dot plots showing Y5R receptor expression in lung cancer brain metastasis (GEO cohort data), various primary tumor xenografts, and metastatic breast and melanoma cancers. **C.** Relative expression of Y5R receptor mRNA in primary brain cells and lung cancer cell lines (CMT167, LL/2, PC9BrM, H2030BrM) as examined by qRT-PCR. normalized to β-Actin (*n=3* independent experiments). **D.** Left: Western blot analysis of Y5R receptor expression in mouse and human lung cancer cell lines (LL/2, CMT167, H2030BrM, PC9BrM) treated with or without recombinant NPY. GAPDH used for normalization (*n=3* independent experiments). Right: Quantitative analysis of band intensities using ImageJ (unpaired two-tailed t-test). **E.** Left: Western blot analysis for y5r receptor expression in ScrKO and y5rKO CMT167 lung cancer cells clones generated using the CRISPR/Cas9 system. GAPDH used for normalization (*n=3* independent experiments). Right: Quantitative analysis of band intensities using ImageJ (unpaired two-tailed t-test). **F.** Cells from panel D were treated with or without recombinant NPY protein (100 nM) and examined for cell proliferation by MTS assay (left) and growth (right) by colony formation assay (*n=3* independent experiments, unpaired two-tailed t-test). **G.** Representative images of hematoxylin and eosin (H&E) on brain metastatic tumor lesions derived from ScrKO and y5rKO normal and low BMI mice (Scale bar: 50 µm). All data are mean ± S.E.M. Source data are provided as a Source Data file.

Supplementary Figure 5

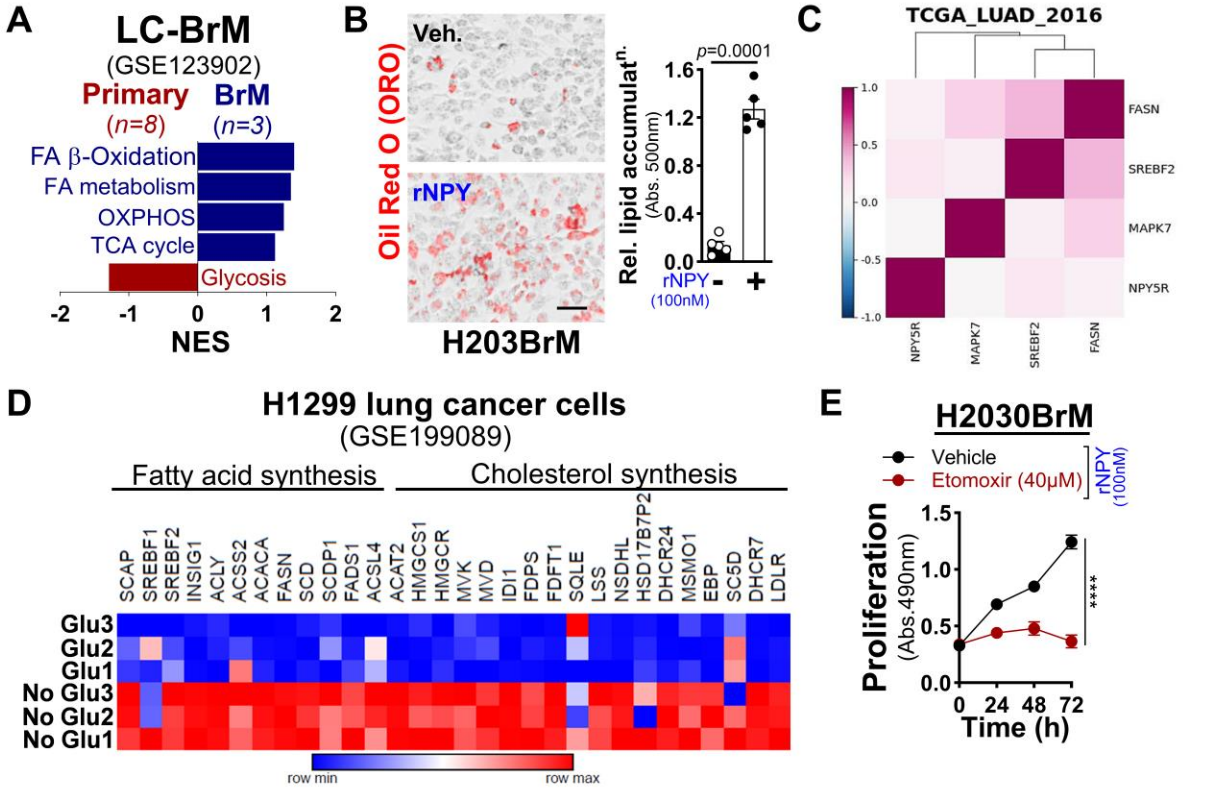

Supplementary Figure 5: Neuronal NPY upregulated lipogenesis to promote tumor cell growth. A. Metabolic GSEA profiling was performed for primary lung (*n=8*) and brain metastatic (*n=3*) tumors from metastatic lung cancer patients using the GEO cohort dataset (GSE123902). Number of genes for each pathway were adapted from Gene Ontology (GO:0006635; GO:0006099; GO:0061621; GO:0046949, GO:0006119, GO:0006631). **B.** Left: Representative images of ORO-stained cancer cells showing lipid accumulation under NPY (100 nM) treatment compared to controls (Scale bar:100 μm). Right: Dot plot representing the quantification of lipid accumulation upon ORO staining in control versus NPY treated cancer cells (*n = 5* individual experiment; unpaired two-tailed t-test). **C.** Correlation analysis between Y5R receptor expression and its downstream activation signaling in primary lung tumors (*n=517*) using TCGA dataset and analyzed via the lung cancer explorer tool as described in Methods. **D.** Heatmap of differentially expressed genes involved in fatty acid and cholesterol synthesis genes in H1299 lung cancer cells cultured under glucose-deprivation conditions (stimulating low BMI condition)

using the GEO dataset (GSE199089). **E.** H2030BrM lung cancer cells were treated with or without CPT1 inhibitor (Etomoxir) and examined for cell proliferation by MTS assay (*n=3* independent experiments, unpaired two-tailed t-test). All data are mean ± S.E.M. Source data are provided as a Source Data file.

Supplementary Figure 6

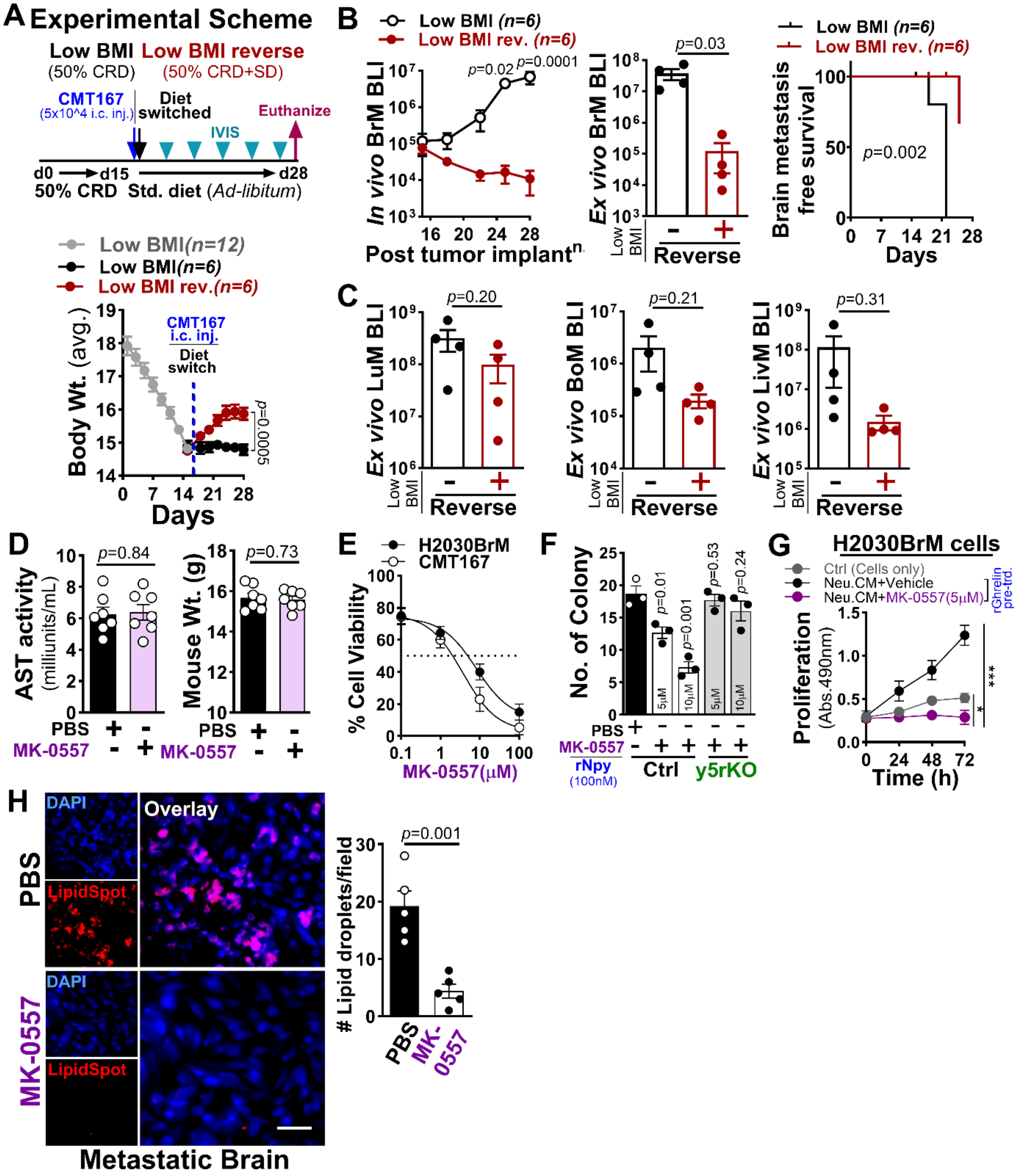

Supplementary Figure 6: Reversal of low BMI or Y5R inhibition attenuates brain metastasis. A. Upper: Schematic of *in vivo* metastasis assay. Female C57BL/6 mice (5-6 weeks) were fed with a 50% calorie restricted diet (12-hr/dark phase) for 15 days, then switched to standard diet *(ad-libitum)* post-implantation of CMT167 cells injection ($5x10^4$; i.c.). Lower: Average body weight curve of each group (two-way ANOVA with Tukey's test). **B.** Left and middle: *In vivo* and *ex vivo* BLI quantification of brain metastasis in control and reversed low BMI mice (*n=6/*group) at the endpoint (two-way ANOVA with Tukey's test (*in vivo*); unpaired two-tailed t-test (*ex vivo*). Right: Kaplan–Meier analysis of brain metastasis-free survival in control and reversed low BMI mice (*n=6/*group) [log-rank (Mantel–Cox test)]. **C.** Representative *ex vivo* BLI images and quantification of lung, liver and bone metastasis in control and reversed low BMI mice (*n=4/*group) at the endpoint (unpaired two-tailed t-test). **D.** Left: Serum aspartate aminotransferase (AST) level of were measured in PBS- or MK-0557-treated mice using an AST activity assay kit (*n*=7/group, unpaired two-tailed t-test). Right: Weight of mice treated with PBS- or MK-0557 (*n=7* mice/group, unpaired two-tailed t-test). **E.** Direct effect of MK-0557 on the viability of H2030BrM and CMT167 cells at varying concentrations, by MTT assay (*n=3* independent experiments). **F.** Control (ScrKO) or y5rKO CMT167 cancer cells were treated overnight with PBS- or MK-0557 (5, 10 µM) in the presence of rNPY and examined for growth by colony formation assay (*n=3* independent experiments, unpaired two-tailed t-test). **G.** H2030BrM lung cancer cells were treated with or without conditioned medium derived from ghrelin-pretreated human primary neurons, in the presence or absence of NPY receptor antagonist (MK-0557). Cell proliferation was assessed by MTS assay (*n=3* independent experiments, unpaired two-tailed t-test). **H.** Right: Representative immunofluorescence images of lipid droplets in metastatic brain tumors derived from PBS- or MK-0557 treated groups by LipidSpot. Lipid droplets were visualized under microscope and measured for 5 random fields (Scale bar:100 µm). Left: Quantification of lipid droplets in PBS- or MK-0557 treated tumor sections using ImageJ (unpaired two-tailed t-test). All data are mean ± S.E.M. Source data are provided as a Source Data file.

Supplementary Figure 7

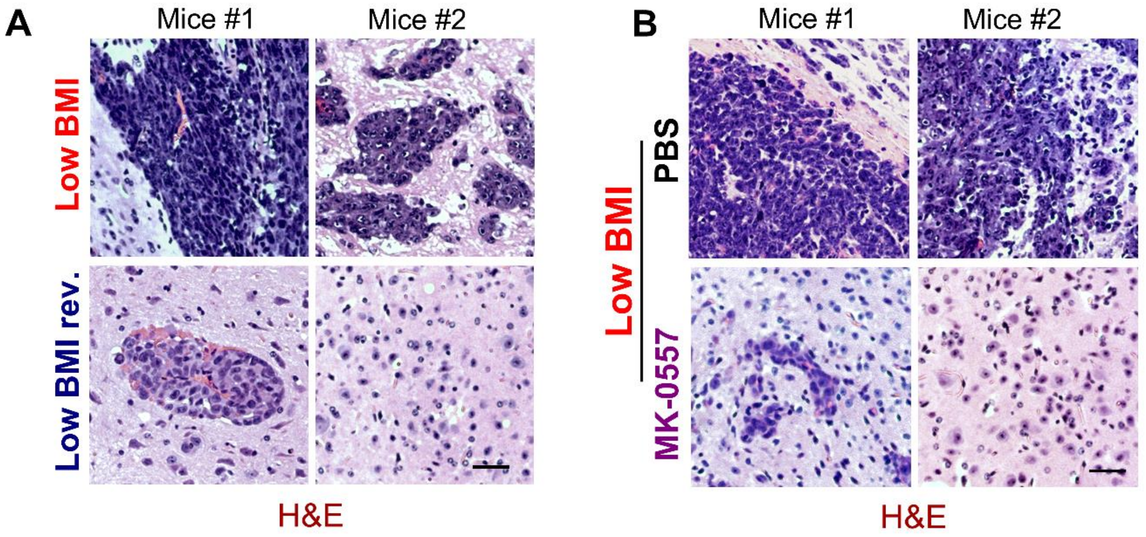

Supplementary Figure 7: Low BMI reversibility and Y5R inhibition mitigate brain metastasis. A. Representative images of hematoxylin and eosin (H&E) on brain metastatic tumor lesions derived from low BMI and low BMI reversed mice from Figure 5, panel A (Scale bar: 50 μm). **B.** Representative images of hematoxylin and eosin (H&E) on brain metastatic tumor lesions derived from PBS and MK-0557-treated low BMI mice from Figure 5, panel G (Scale bar: 50 μm).

Supplementary Figure 8

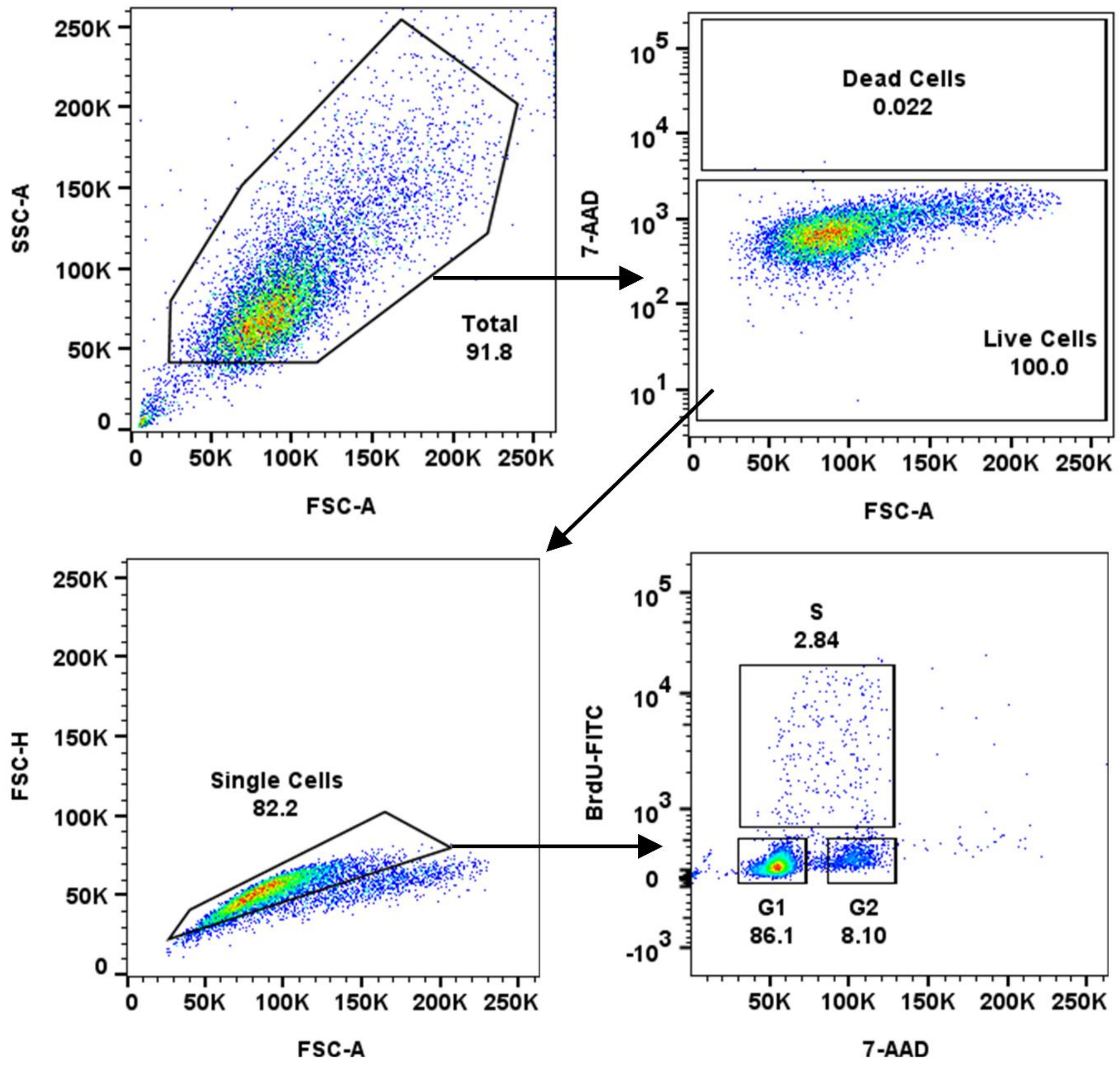

Supplementary Figure 8: Representative gating strategy for detection of BrdU+ proliferating cells by flow cytometry related to Figure 2L.

Supplementary Table 1

Normal mice (n=7)				Low BW mice (n=8)			
Days	Avg.BW (g)	Avg.Height (cm)	BMI(g/cm^2)	Days	Avg.BW (g)	Avg.Height (cm)	BMI(g/cm^2)
1	18.20	8.33	0.262	1	18.19	8.38	0.259
3	18.60	8.45	0.26	3	17.86	8.35	0.256
5	18.69	8.41	0.264	5	17.45	8.4	0.247
7	18.77	8.37	0.267	7	17.05	8.41	0.241
9	19.00	8.42	0.267	9	16.63	8.43	0.234
11	19.29	8.32	0.278	11	16.25	8.46	0.227
13	19.21	8.46	0.268	13	15.91	8.5	0.22
15	19.21	8.44	0.269	15	15.64	8.41	0.221
17	19.53	8.51	0.269	17	15.46	8.52	0.213
19	19.57	8.48	0.272	19	15.34	8.47	0.213
21	19.67	8.54	0.269	21	15.68	8.55	0.214
23	19.67	8.52	0.27	23	15.74	8.57	0.214
25	19.99	8.57	0.272	25	15.80	8.59	0.214
27	20.22	8.6	0.273	27	15.75	8.61	0.212
29	19.92	8.55	0.272	29	15.71	8.5	0.217
31	20.14	8.62	0.271	31	15.63	8.53	0.214
33	20.30	8.64	0.272	33	15.77	8.59	0.213
35	20.36	8.6	0.275	35	15.71	8.62	0.211

Supplementary Data 1: Raw BMI values of normal and low BMI mice used in the study. Data represent individual BMI measurements corresponding to the groups shown in Supplementary Figure 2C.

Supplementary Table 2

ID	Gene	logFC	p-Value	Brain/Blood secretory
ENSG00000107742	SPOCK2	35.91	0.0356	yes
ENSG00000259384	GH1	22.95	0.0188	yes
ENSG00000122585	NPY	16.22	0.0455	yes
ENSG00000125730	C3	14.57	0.0120	yes
ENSG00000172179	PRL	14.05	0.0178	yes
ENSG00000157150	TIMP4	12.44	0.0131	yes
ENSG00000213398	LCAT	11.05	0.0079	yes

Supplementary Data 2: Identification of low BMI-specific brain secretory factors using GTEx and HPA database screening related to Figure 2F.

Supplementary Table 3

Primer name	Sequence (5' to 3')
hGHSR-F	GCTGTCGTGGGTGCCTCGCTC
hGHSR-R	GCCACCCGGTACTTCTTGGACATGATG
hSPOCK2-F	GCAGCCCTGGCCGAAGGCG
hSPOCK2-R	GCTGGGCCATGTGGCAGGGCT
hGH1-F	GCCTGCTCTGCCTGCCCTGG
hGH1-R	GCAGCAGGGAGATGCGGAGCA
hNPY-F	GCCCGACAGCATAGTACTTGCCG
hNPY-R	GCGTCCTCGCCCGGGTTGTCC
hC3-F	GCGCAACAAGTTCGTGACCGTGC
hC3-R	GCAAGACGCCAAGCTGGTTCTGAG
hPRL-F	GCTGCTGCTGGTGTCAAACCTGC
hPRL-R	GCAAGGGAAGAAGTGTGGCAGCTG
hTIMP4-F	GCCGTCCAGTCCCCCAGACCTCA
hTIMP4-R	GCAGGGTCTGCACTGGCCGGAA
hLCAT-F	GCTGGAATGGGGCCGCCCGG
hLCAT-R	GCGGATCTGGACACCAGGGGCG
hNPY5R-F	GCCTGGAAATTCTGATGGTGGGAGATAC
hNPY5R-R	GCAAGACAGACGTCAGTGTGAAAGGTG
hSREBP2-F	ATCTGGATCTCGCCAGAGG
hSREBP2-R	CCAGGCAGGTTTGTAGGTTG
hFFASN-F	AAGGACCTGTCTAGGTTTGATGC
hFFASN-R	TGGCTTCATAGGTGACTTCCA
hCPT1A-F	GCTCGCCGCTCGTTCACTCCAC
hCPT1A-R	GCTTGCCGGGTACACGCCAGTG
hActin-F	TGAGACCTTCAACACCCCAGCCATG
hActin-R	CGTAGATGGGCACAGTGTGGGTG
mGhsr-F	GGACCAGAACCACAAACAGACA
mGhsr-R	CAGCAGAGGATGAAAGCAAACA
mGH1-F	GCAGGAGCCTTGGGGTCGAG
mGH1-R	GCTGTCCCTCGGGAATGTAGGC
mNPY-F	GTGTGTTTGGGCATTCTGGC
mNPY-R	GGGGCGTTTTCTGTGCTTTC
mPRL-F	GCTGTGGTTCTCTCAGGCCATCTTG
mPRL-R	GCCAGGGAAGAAGTGGGGCAG
mNpy5r-F	GCCCCTCCTTCCCACCGCC
mNpy5r-R	GCTGCCTCTATAGTCCTCCCAGAC
mSrebp2-F	GCTGGTGGCCTTTGTTTCGGCGG
mSrebp2-R	CGCTGGGCCTGTACCGTCTGC
mFasn-F	GGAGGTGGTGATAGCCGGTAT
mFasn-R	TGGGTAATCCATAGAGCCCAG
mCpt1a-F	GCATCGATCTCCGCCTGAGCC
mCpt1a-R	CGGCTCATTTTGCCGTGCTCTGC
mActin-F	AGGGTGTGATGGTGGGAATG
mActin-R	GGTACGACCAGAGGCATACA

Supplementary Data 3: Table showing the list of primers used in this study.
