# Supplementary material for: Ghrelin-induced neuronal NPY promotes brain metastasis in lung cancer patients with low BMI
Source: Nat Commun. 2025 Jul 1;16:5608. doi: 10.1038/s41467-025-60730-4 (PMC12218320; doi:10.1038/s41467-025-60730-4)
Supplement: Supplementary file 2 — Reporting Summary [file 41467_2025_60730_MOESM2_ESM.pdf]

nature portfolio

Corresponding author(s): Kounosuke Watabe

Last updated by author(s): Apr 23, 2025

Reporting Summary

Nature Portfolio wishes to improve the reproducibility of the work that we publish. This form provides structure for consistency and transparency in reporting. For further information on Nature Portfolio policies, see our Editorial Policies and the Editorial Policy Checklist.

Statistics

For all statistical analyses, confirm that the following items are present in the figure legend, table legend, main text, or Methods section.

n/a	Confirmed	
☐	☒	The exact sample size (*n*) for each experimental group/condition, given as a discrete number and unit of measurement
☐	☒	A statement on whether measurements were taken from distinct samples or whether the same sample was measured repeatedly
☐	☒	The statistical test(s) used AND whether they are one- or two-sided<br>*Only common tests should be described solely by name; describe more complex techniques in the Methods section.*
☒	☐	A description of all covariates tested
☐	☒	A description of any assumptions or corrections, such as tests of normality and adjustment for multiple comparisons
☐	☒	A full description of the statistical parameters including central tendency (e.g. means) or other basic estimates (e.g. regression coefficient) AND variation (e.g. standard deviation) or associated estimates of uncertainty (e.g. confidence intervals)
☐	☒	For null hypothesis testing, the test statistic (e.g. *F*, *t*, *r*) with confidence intervals, effect sizes, degrees of freedom and *P* value noted<br>*Give P values as exact values whenever suitable.*
☒	☐	For Bayesian analysis, information on the choice of priors and Markov chain Monte Carlo settings
☒	☐	For hierarchical and complex designs, identification of the appropriate level for tests and full reporting of outcomes
☐	☒	Estimates of effect sizes (e.g. Cohen's *d*, Pearson's *r*), indicating how they were calculated

Our web collection on statistics for biologists contains articles on many of the points above.

Software and code

Policy information about availability of computer code

Data collection	Immunofluorescence images was acquired using Keyence All-in-one fluorescence microscope (BZ-X700). Flow cytometry data was acquired using BD FACS Canto analyzer. Bioluminescence was acquired using Xenogen IVIS imaging system (Caliper Life Science) version 4.7.3 software. Immunoblotting images were captured by Amersham Imager 600. ImageJ (version 1.53c) was used for image quantification analysis, Microsoft excel (version 2409) was used to create supplementary and source data file.
Data analysis	Flow cytometry data was analyzed with pre-installed FACS Diva software. Bioluminescence was analyzed using Xenogen IVIS imaging system (Caliper Life Science) version 4.7.3 software. ImageJ (version 1.53c) was used for image quantification analysis. Statistical analysis was performed by GraphPad Prism (version 10). Living image software was used for analyzing images.

For manuscripts utilizing custom algorithms or software that are central to the research but not yet described in published literature, software must be made available to editors and reviewers. We strongly encourage code deposition in a community repository (e.g. GitHub). See the Nature Portfolio guidelines for submitting code & software for further information.

Data

Policy information about availability of data

All manuscripts must include a data availability statement. This statement should provide the following information, where applicable:

- Accession codes, unique identifiers, or web links for publicly available datasets
- A description of any restrictions on data availability
- For clinical datasets or third party data, please ensure that the statement adheres to our policy

Previously published micro-array, RNA-seq. and proteomic data that were reanalyzed in this study are available in GEO under accession codes GSE200563, GSE123902, GSE48433, GSE14020, GSE48433, GSE199089, GSE14020, GSE50493 (https://www.ncbi.nlm.nih.gov/gds) or at PRIDE - Proteomics Identification Database under accession codes PXD027259 (https://central.proteomexchange.org/PXD027259). Human protein atlas, GTEx database, Kaplan–Meier survival analysis, TCGA lung, Gene Ontology referenced during the study are available in a public repository from https://www.proteinatlas.org/; https://www.gtexportal.org/home/; http://kmplot.com/analysis/index.php?p=service; https://lce.biohpc.swmed.edu/lungcancer/; http://geneontology.org/ website. Source data are provided with this paper including supplementary files and Data availability statementis is included in the manuscript.

Research involving human participants, their data, or biological material

Policy information about studies with human participants or human data. See also policy information about sex, gender (identity/presentation), and sexual orientation and race, ethnicity and racism.

Reporting on sex and gender	Formalin-fixed paraffin-embedded (FFPE) tumor tissues from lung cancer brain metastatic patients with a BMI history were obtained from the Tumor Tissue and Pathology Shared Resource at Wake Forest Baptist Comprehensive Cancer Center. All samples were deidentified and collected in accordance with the Wake Forest School of Medicine IRB-approved protocol (IRB00107587). Informed consent was obtained from all participants. Demographic details, including BMI, age, gender, race, and smoking status were provided in the source data file.
Reporting on race, ethnicity, or other socially relevant groupings	Patient samples were selected regardless of their race. Race is not relevant to this study. All demographic details were provided in the source data file.
Population characteristics	Not applicable
Recruitment	Formalin-fixed paraffin-embedded (FFPE) tumor tissues were obtained from lung cancer patients with brain metastases and documented BMI history, through the Tumor Tissue and Pathology Shared Resource at Wake Forest Baptist Comprehensive Cancer Center. All samples were deidentified and collected under an IRB-approved protocol (IRB00107587) by the Wake Forest School of Medicine. Informed consent was obtained from all participants prior to tissue collection. Relevant demographic information, including BMI, age, gender, race, and smoking status, is provided in the source data file.
Ethics oversight	All samples used in this study were collected with the approval of the institutional review boards of Wake Forest Baptist Comprehensive Cancer Center. Written informed consent was obtained from all participants prior to sample collection.

Note that full information on the approval of the study protocol must also be provided in the manuscript.

Field-specific reporting

Please select the one below that is the best fit for your research. If you are not sure, read the appropriate sections before making your selection.

[x] Life sciences   [ ] Behavioural & social sciences   [ ] Ecological, evolutionary & environmental sciences

For a reference copy of the document with all sections, see nature.com/documents/nr-reporting-summary-flat.pdf

Life sciences study design

All studies must disclose on these points even when the disclosure is negative.

Sample size	Sample size was calculated based on previous experiment done (Liu Y, 2019, PMID: 30725231; Xing F, 2018, PMID: 30026327). Using power analysis, the anticipated means and standard error of means were included to determine the sample size that is expected to yield a power of approximately 80 percent using a p-value of 0.05.
Data exclusions	No data were excluded from the analysis.
Replication	The number of experimental replicates or independently performed experiments for each specific result is indicated in the respective Figure Legends. Representative data shown are from experiments repeated independently at least three times with similar outcomes.
Randomization	For cell experiments, all cells in each experiment were from the same pool of parental and brain-tropic cells. All mice were age- and sex-matched (female mice) and then randomized into different experimental groups. All animals were maintained in the same environment and handled by the same procedure.
Blinding	All data collected by objective instruments, such as plate readers, qPCR cyclers, microscopy software, flow cytometers, animal IVIS systems, and western blotting, the investigators were blinded to group allocation during data collection. Laboratory personnel were blinded to animal

randomization to drug treatment as well as during the data analysis from each individual mice. Data analysis was performed by the Biostatistian who were blinded to experimental groups.

Reporting for specific materials, systems and methods

We require information from authors about some types of materials, experimental systems and methods used in many studies. Here, indicate whether each material, system or method listed is relevant to your study. If you are not sure if a list item applies to your research, read the appropriate section before selecting a response.

Materials & experimental systems

n/a	Involved in the study
☐	☒ Antibodies
☐	☒ Eukaryotic cell lines
☒	☐ Palaeontology and archaeology
☐	☒ Animals and other organisms
☒	☐ Clinical data
☒	☐ Dual use research of concern
☒	☐ Plants

Methods

n/a	Involved in the study
☒	☐ ChIP-seq
☐	☒ Flow cytometry
☒	☐ MRI-based neuroimaging

Antibodies

Antibodies used

Immunoblotting:
anti-PKCα antibody (1:1000, Cell Signaling, #3579S)
anti-pPKCα antibody (1:1000, Cell Signaling, #34903S)
anti-ERK5 antibody (1:1000, Cell Signaling, #D23E9)
anti-pERK5 antibody (1:10000, ThermoFisher, #44-612G)
anti-FoxO1 antibody (1:1000, Cell Signaling, #2880T)
anti-AMPKα antibody (1:1000, cell Signaling, #2532S)
anti-pAMPKα antibody (1:1000, cell Signaling, #2535T)
anti-SIRT1 antibody (1:1000, cell Signaling, #8469T)
anti-p53 antibody (1:1000, cell Signaling, #2527T)
anti-pCREB antibody (1:1000, Cell Signaling, #9198T)
anti-pSTAT3 antibody (1:1000, Cell Signaling, #9145S)
anti-PPARγ antibody (1:1000, Cell Signaling, #2443)
anti-FASN antibody (1:1000, cell Signaling, #3180T)
anti-SREBP2 antibody (1:500, Novus Biologicals, #NB100-74543)
anti-pSREBP2 antibody (1:500, ThermoFisher, #PA5-106042)
anti-NPY antibody (1:500, Cell Signaling, #11976T)
anti-NPY5R antibody (1:500, ThermoFisher, #PA5-106850)
anti-BSX antibody (1:100, MyBioSource.com, #MBS9609602)
anti-GAPDH antibody (1:10000, Cell Signaling, #2374)
Horseradish peroxidase-conjugated anti-mouse (Cell Signaling Technology, #7076) or anti-rabbit (Bio-Rad, #1706515) secondary antibodies.

Validation

The following antibodies were quality-checked and validated based on the information provided on the manufacturers' websites:
https://www.cellsignal.com/products/primary-antibodies/pkca-d7e6e-rabbit-mab/59754
https://www.thermofisher.com/antibody/product/Phospho-PKC-alpha-Thr638-Antibody-Polyclonal/44-962G
https://www.cellsignal.com/products/primary-antibodies/erk5-d23e9-rabbit-mab/3552
https://www.thermofisher.com/antibody/product/Phospho-ERK5-Thr218-Tyr220-Antibody-Polyclonal/44-612G
https://www.cellsignal.com/products/primary-antibodies/foxo1-c29h4-rabbit-mab/2880
https://www.cellsignal.com/products/primary-antibodies/ampka-antibody/2532
https://www.cellsignal.com/products/primary-antibodies/phospho-ampka-thr172-40h9-rabbit-mab/2535
https://www.cellsignal.com/products/primary-antibodies/sirt1-1f3-mouse-mab/8469
https://www.cellsignal.com/products/primary-antibodies/p53-7f5-rabbit-mab/2527
https://www.cellsignal.com/products/primary-antibodies/phospho-creb-ser133-87g3-rabbit-mab/9198
https://www.cellsignal.com/products/primary-antibodies/phospho-stat3-tyr705-d3a7-xp-rabbit-mab/9145
https://www.cellsignal.com/products/primary-antibodies/pparg-81b8-rabbit-mab/2443
https://www.cellsignal.com/products/primary-antibodies/fatty-acid-synthase-c20g5-rabbit-mab/3180
https://www.novusbio.com/products/srebp2-antibody_nb100-74543?srsltid=AfmBOoovAD7-rKhW6aRvnk-CmHNs5zC0GP1CZX1AG8iV3WCtGuRwN8cd
https://www.thermofisher.com/antibody/product/Phospho-SREBP2-Ser455-Antibody-Polyclonal/PA5-106042
https://www.cellsignal.com/products/primary-antibodies/neuropeptide-y-d7y5a-xp-rabbit-mab/11976
https://www.thermofisher.com/antibody/product/NPY5R-Antibody-Polyclonal/PA5-106850
https://www.mybiosource.com/polyclonal-human-mouse-rat-antibody/bsx/9609602
https://www.cellsignal.com/products/primary-antibodies/gapdh-d16h11-xp-rabbit-mab/5174

https://www.cellsignal.com/products/secondary-antibodies/anti-mouse-igg-hrp-linked-antibody/7076
https://www.bio-rad.com/en-us/sku/1706515-goat-anti-rabbit-igg-h-l-hrp-conjugate?ID=1706515
Every antibody used in this study had been previously validated by the manufacturer.

Eukaryotic cell lines

Policy information about cell lines and Sex and Gender in Research

Cell line source(s)	Human lung cancer brain-tropic cell lines H2030BrM and PC9BrM were a kind gift from Dr. Massague (Memorial Sloan-Kettering Cancer Center). Mouse lung carcinoma cell lines, LL/2 and CMT167, were purchased from American Type Culture Collection (ATCC) and MilliporeSigma. iPSC-derived GABAergic human primary neurons were purchased from BrainXell. E6/E7/hTERT immortalized human astrocyte, UC1 were a kind gift from Dr Russell Piper (University of California-San Francisco) and Human microglial cell line HMC3 was purchased from ATCC.
Authentication	All cell lines were authenticated from the ATCC, MilliporeSigma, BrainXell and from original provider (https://pubmed.ncbi.nlm.nih.gov/19576624/; https://pubmed.ncbi.nlm.nih.gov/11431323/).
Mycoplasma contamination	All cell lines used in this study were tested for mycoplasma contamination by PCR method. All cell lines used in this study were negative for mycoplasma.
Commonly misidentified lines (See ICLAC register)	None of cell lines used in this study are listed by ICLAC.

Animals and other research organisms

Policy information about studies involving animals; ARRIVE guidelines recommended for reporting animal research, and Sex and Gender in Research

Laboratory animals	All animal experiments were done in accordance with the U.S. National Institutes of Health Animal Protection Guidelines, and the protocol was approved by the Wake Forest Baptist Health Institutional Animal Care and Use Committee. All mice (C57BL/6; 5-6 weeks' age; female) were housed individually and were maintained in a specific pathogen free unit on a 12-hour light: 12-hour dark cycle with either ad-libitum or 50% calorie restriction with time-restricted feeding and access to water. The animal rooms were provided with 100% fresh, HEPA filtered air at 10-15 air changes per hour. Room temperatures were controlled by reheat units within each room, and were maintained within the range of 70°F ± 2° F. The humidity levels were controlled and maintained between 30-70%.
Wild animals	The study did not involve wild animals.
Reporting on sex	All animals used in the study were female as described in Methods based on the data indicated in human sample analysis (Figure 1A-C; Supplementary Figure 1A-B) that demonstrate general impact on both sexes/gender.
Field-collected samples	No field-collected samples were used in this study.
Ethics oversight	All experiments were reviewed and approved by the IACUC of Wake Forest Baptist Health Institutional Animal Care and Use Committee under the protocol A21-171

Note that full information on the approval of the study protocol must also be provided in the manuscript.

Plants

Seed stocks	N/A
Novel plant genotypes	N/A
Authentication	N/A

Flow Cytometry

Plots

Confirm that:

- [x] The axis labels state the marker and fluorochrome used (e.g. CD4-FITC).
- [x] The axis scales are clearly visible. Include numbers along axes only for bottom left plot of group (a 'group' is an analysis of identical markers).
- [ ] All plots are contour plots with outliers or pseudocolor plots.
- [x] A numerical value for number of cells or percentage (with statistics) is provided.

Methodology

Sample preparation	For BrdU incorporation assay, cancer cells were treated with or without recombinant NPY or ghrelin with the desired concentration as indicated in the figure. BrdU incorporation assay was performed using FITC BrdU Flow kit (BD Biosciences) according to the manufacturer's instructions. Briefly, cells were pulsed with 10 µM BrdU and stained with anti-BrdU antibody following DNA digestion. Cells were then stained with nuclear stain 7-AAD, and data was recorded and analyzed by flow cytometry using the FACS Canto II flow cytometer and FACSDiva software (BD Biosciences).
Instrument	BD FACS Canto II Flow Cytometer (BD Biosciences).
Software	BD FACSDiva vrsion 8.01, FlowJo version 10.8.1
Cell population abundance	Dead cells and doublets was excluded on the basis of forward and side scatter and fixable Live/Dead dye.
Gating strategy	Briefly, cells were first gated by FSC and SSC, followed by dead cell exclusion with respective fluorochrome, followed by FSC-H and FSC-A for single cell populations. Gating strategy have been provided in Supplementary Information File.

- [x] Tick this box to confirm that a figure exemplifying the gating strategy is provided in the Supplementary Information.
